# RACK7 Interacts with PRC2 Complex to Regulate Astrocyte Development

**DOI:** 10.1002/advs.202416350

**Published:** 2025-03-24

**Authors:** Fangfang Jiao, Tianxiang Tang, Bowen Wang, Shengfei He, Yue Zhang, Li Dong, Bo Xu, Ying Liu, Ping Zhu, Rui Guo

**Affiliations:** ^1^ Center for Medical Research and Innovation Shanghai Pudong Hospital Fudan University Pudong Medical Center and the Shanghai Key Laboratory of Medical Epigenetics the International Co‐laboratory of Medical Epigenetics and Metabolism Ministry of Science and Technology Institutes of Biomedical Sciences Fudan University Shanghai 200032 China; ^2^ State Key Laboratory of Eye Health Department of Ophthalmology Shanghai Ninth People's Hospital Shanghai Jiao Tong University School of Medicine Shanghai 200011 China; ^3^ Center for Excellence in Brain Science and Intelligence Technology (Institute of Neuroscience) Chinese Academy of Sciences 320 Yue Yang Road Shanghai 200031 China; ^4^ Institutes of Biomedical Sciences Fudan University Shanghai 200032 China; ^5^ Guangdong Cardiovascular Institute Guangdong Provincial People's Hospital Guangdong Academy of Medical Sciences Guangzhou Guangdong 510100 China; ^6^ Department of Animal Dairy and Veterinary Sciences Utah State University Logan UT 84322 USA; ^7^ Aging and Longevity Institute & Institute of Biological Science Zhongshan Hospital Fudan University Shanghai 200032 China

**Keywords:** Astrocyte development, H3K27me3, PRC2 complex, RACK7, Wnt signaling pathway, ZMYND8

## Abstract

Dysregulation of epigenetic mechanisms plays a crucial role in brain development and disease. Emerging largely evidence suggests that Receptor for Activated C‐kinase 7 (RACK7), an epigenetic reader protein, may play a role in brain development and neural developmental disease, but in vivo explorations are still lacking. Here, a *Rack7* conditional knock‐out mouse model is established and shows that *Rack7*‐deficient mice exhibit overt developmental defects associated with aberrant astrocyte development. Mechanistically, it is found that RACK7 interacts with the histone H3 lysine 27 (H3K27) methyltransferase, i.e., the Polycomb Repressive Complex 2 (PRC2) complex, to establish the genomic locations of Suppressor of Zeste 12 homolog (SUZ12) and H3K27 methylation. Deletion of *Rack7* in astrocytes leads to a remarkable decrease of H3K27me3 chromatin localization genome‐wide. Furthermore, RACK7 works together with H3K27me3 to prevent overactivation of the Wnt signaling pathway and other astrocyte differentiation genes are found. Collectively, this study provides new insights into the cellular and molecular mechanisms underlying brain development regulated by RACK7.

## Graphical abstract

The study investigates the role of RACK7 in brain development using a conditional knockout mouse model. RACK7 deficiency results in developmental defects and abnormal astrocyte development by disrupting H3K27me3 chromatin binding. The underlying mechanism involves RACK7 interacting with the PRC2 complex to regulate the genomic localization of SUZ12 and H3K27me3.

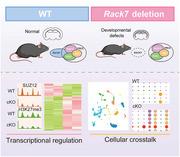

## 1 Introduction

Chromatin is composed of repeating units of nucleosomes, which includes a 147 bp segment of DNA and two copies each of four core histone proteins H2A, H2B, H3, and H4.^[^ ^1^ ^]^ Post‐translational modifications (PTMs) in histone are dynamically added or removed by enzymes that serve as “writer” or “eraser”, respectively, and are recognized by effector proteins serve as “reader”. Dysregulation of histone modifications plays an important role in various biological processes, including nervous system development and disease.^[^ ^2^, ^3^ ^]^ Advances in DNA sequencing technologies also led to the identification of genetic mutations in many chromatin regulators. For example, H3 lysine 27 trimethylation (H3K27me3) has been found to be associated with repressive transcription and function in early embryonic development.^[^ ^4^ ^]^ H3K27me3 is mediated by the PRC2 complex, whose catalytic component Enhancer of Zeste Homolog 2 (EZH2) has been found to regulate neuronal morphogenesis, synaptic plasticity, and cognitive behavior in mice through H3K27me3 modification.^[^ ^5^ ^]^ However, studies of dysregulation of PTMs in nervous system development are more focused on the roles of “writers” and “erasers”, much less on “readers”. Here, we provide new insight into the epigenetic mechanisms that regulate brain development, which is mediated by the chromatin reader *Rack7*.

RACK7, also known as ZMYND8 (Zinc finger MYND‐type containing 8) and PRKCBP1 (Protein Kinase C Binding Protein 1), is a chromatin‐binding protein involved in transcriptional regulation and histone/DNA recognition. RACK7 has been shown to read dual histone modifications, such as the H3.1K36 dimethylation and H4K16 acetylation (H3.1K36me2‐H4K16ac) and H3K4 monomethylation/H3K14 acetylation (H3K4me1‐H4K14ac).^[^ ^6^, ^7^ ^]^ Moreover, our previous study found that RACK7 recognizes H3.3G34R mutation which is detected in childhood pediatric patients, that was further validated by an Nuclear Magnetic Resonance (NMR) structure.^[^ ^8^, ^9^ ^]^ Functionally, RACK7 works as a transcriptional co‐repressor interacting with several repressor complexes. The MYND domain of RACK7 interacts with the Nucleosome Remodeling and Deacetylase Complex‌‌ (NuRD) , which participates in the DNA damage response and regulation of transcription.^[^ ^10^ ^]^ RACK7 also interacts with Lysine Demethylase 5C (KDM5C), a histone demethylase of H3K4me3, to suppress enhancer overactivation and contribute to breast cancer.^[^ ^11^ ^]^ Finally, RACK7 works with the H3K4 demethylase Lysine Demethylase 5D (KDM5D) and antagonizes the expression of metastasis‐linked genes in prostate cancer cells.^[^ ^7^ ^]^

In addition to a role in tumorigenesis, emerging evidence also suggests a function of RACK7 in nervous system development. In *Xenopus*, RACK7 interacts with REST corepressor 2 (RCOR2), which functions as a transcriptional repressor involved in the regulation of neural differentiation.^[^ ^12^ ^]^ RACK7 forms a homodimer that preferentially associates with the P‐TEFb complex to activate transcription of genes required for all‐trans retinoic acid (ATRA)‐mediated differentiation of neuronal precursor cells.^[^ ^13^ ^]^ Our previous study showed that RACK7 also represses genes associated with cell differentiation in the H3.3G34R‐containing pediatric glioblastoma cell line.^[^ ^8^ ^]^ Interestingly, *de‐novo* mutations of ZMYND family proteins, including RACK7,^[^ ^14^ ^]^ BS69 (aka Zinc finger MYND‐type containing 11, ZMYND11)^[^ ^15^ ^]^ and ZMYND5 (aka Deformed Epidermal Auto‐regulatory Factor 1, DEAF1),^[^ ^16^ ^]^ have been found in neurodevelopmental diseases, such as intellectual disability. These studies collectively suggest that RACK7 may have functions in central nervous (CNS) development. While much work on RACK7 has been done in cell lines, the in vivo function and the underly mechanisms for RACK7 are still uncertain.

In this study, we generated a *Rack7* conditional knockout (cKO) mouse model to interrogate the potential role of RACK7 in brain development and behavior, and to explore the underlying cellular and molecular mechanisms of RACK7 in this developmental process.

## 2 Results

### 2.1 Generation of a *Rack7* Conditional Knockout Mouse Model

**Figure 1:**
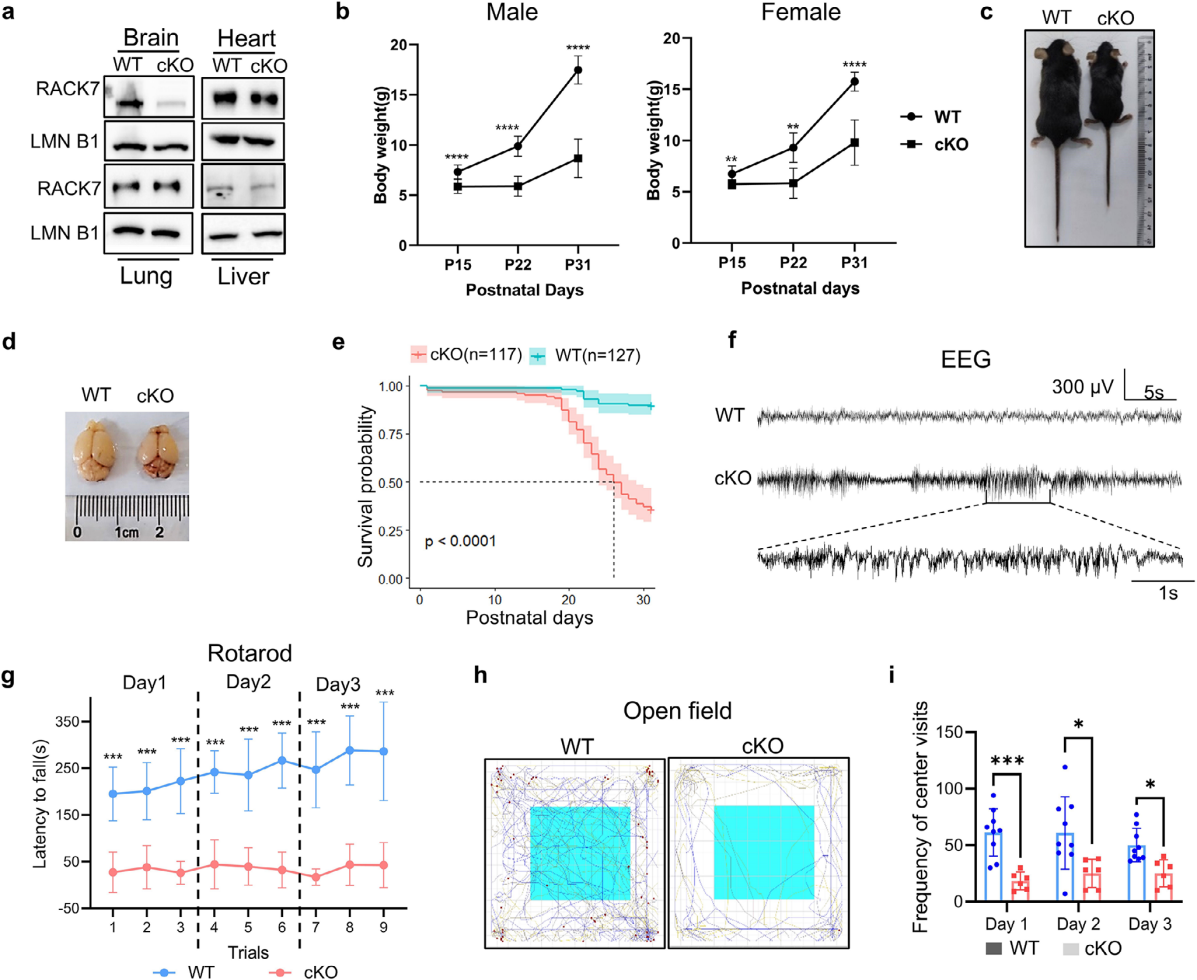
*Rack7*‐cKO mice exhibit developmental defects. a) Immunoblots of mouse tissues from wild‐type (WT) and *Rack7‐*cKO mice. b) Both male and female *Rack7*‐cKO mice exhibited lower body weights at postnatal days P15, P22 and P31, with more than 10 mice per group (number of animals (*n)* > 10). c) Body size comparison between WT and *Rack7*‐cKO mice. d) Brain size comparison between WT and *Rack7*‐cKO mice. e) Kaplan‐Meier survival curves of WT and *Rack7*‐cKO mice. f) *Rack7*‐cKO mice showed a higher incidence of spontaneous seizures compared to *Rack7*‐WT mice during a 47‐hour EEG recording period. g) Latency to fall from an accelerating rotarod in WT and *Rack7*‐cKO mice, WT, *n* = 13; cKO, *n* = 7. Nine trials were conducted over 3 consecutive days. h) Representative movement tracing from the open field test for WT (*n* = 9) and *Rack7*‐cKO (*n* = 6) mice. i) The frequency of center crosses in an open field arena over a 30‐minute trial on 3 consecutive days (WT, *n* = 9; cKO, *n* = 6). Data in b, g, i are presented as the mean ± SD, statistical significance was determined using two‐way ANOVA; *p ≤ 0.05, **p ≤ 0.01, ***p ≤ 0.001, ****p ≤ 0.0001, n.s. represents not significant.

Cortex and hippocampus both originate from the dorsal forebrain and are vital for regulating cognitive abilities, motor skills, memory, and emotional perception. To explore the function of *Rack7* in cortex and hippocampus development process and related behavior alterations, we deleted *Rack7* in these regions using a human glial fibrillary acid protein (GFAP) promoter‐driven Cre (hGFAP‐Cre), which is expressed at embryonic day (E) 13.5, and targeting multi‐potential neural stem cells which differentiated into parts of neuronal and glial cells (Figure S1a, Supporting Information).^[^ ^17^, ^18^ ^]^ Homozygous conditional deletion of *Rack7* (hGFAP‐cre:: *Rack7* ^fl/fl^, referred as cKO) was verified by PCR (compared with wildtype mice (hGFAP‐cre:: *Rack7* ^+/+^, referred to as WT) (Figure S1b, Supporting Information). Immunoblot analysis confirmed selective RACK7 protein loss specifically in the brain but not in other tissues (**Figure** **1**a). We found that *Rack7*‐cKO male and female mice exhibited smaller body size and reduced body weight (Figure 1b,c; *p* < 0.01, two‐way ANOVA). Consistently, *Rack7*‐cKO mice also showed reduced brain size (Figure 1d). However, no significant alterations in the body/brain weight and other phenotypes were observed in the neonatal cKO mice compared with their littermate WT. Moreover, Kaplan‐Meier survival analysis of 127 WT and 117 *Rack7*‐cKO mice showed increased death in cKO mice (*p* < 0.0001) with a medium survival at about postnatal day (P) 25 (Figure 1e). Meanwhile, we observed spontaneous seizures in *Rack7*‐cKO mice during a 47‐hour Electroencephalogram (EEG) recording period, which indicates altered brain function (Figure 1f).

Analysis of the surviving *Rack7*‐cKO mice (3‐4 weeks of age) found that they also exhibited altered behaviors before death, notably the motor coordination and motor learning of *Rack7*‐cKO mice based on the rotarod task assay. Specifically, cKO mice exhibited overtly shorter latencies to fall over 9 trials in three consecutive days, and the duration of cKO mice on the rotarod was not prolonged through learning, which suggested serious motor decay due to *Rack7* deletion (Figure 1g). To determine whether the motor coordination deficits observed in *Rack7*‐cKO mice were accompanied by alterations in their spontaneous locomotor activity, we employed an open‐field test (Figure 1h). *Rack7*‐cKO mice displayed similar activity levels as the WT mice in the open field (2‐way ANOVA) (Figure S1c, Supporting Information), but the *Rack7*‐cKO mice crossed the center of the open field less than WT, especially in the first day (2‐way ANOVA, Day 1 *p* < 0.001, Day 2 and 3 *p* < 0.05), suggesting that *Rack7*‐cKO mice may have an anxiety‐related phenotype (Figure 1i). Besides, we also assessed the learning and memory abilities of *Rack7*‐cKO mice using the Y‐maze. The number of spontaneous alternations was found to be comparable between *Rack7*‐cKO and WT mice (Figure S1d, Supporting Information). Furthermore, in novel object exploration, *Rack7*‐cKO mice did not exhibit a preference for the novel object and spent an equal amount of time with WT mice during both the habituation and exploration periods (Figure S1e, Supporting Information).

Collectively, our findings suggest that deletion *Rack7* in the mouse brain leads to developmental defects, consisting of growth retardation, motor coordination defects, anxiety‐related behaviors, and premature death. The developmental delay in *Rack7*‐cKO mice began to emerge between P7 and P14, to be significantly detectable around P14, and was further exacerbated throughout postnatal development. Notably, except for the death phenotype, most of the other phenotypes were observed in intellectual disability patients with de‐novo RACK7 variants.^[^ ^14^ ^]^

### 2.2 RACK7 Deletion is Primarily Observed in the Cerebral Cortex and Hippocampus

To determine whether loss of *Rack7* changed the overall brain architecture, we performed Nissl staining and found no gross abnormalities in the cytoarchitecture of the postnatal *Rack7*‐cKO brain (one month old), despite the reduced brain size (Figure S2a, Supporting Information). We further stained brain tissue sections for RACK7 and observed that RACK7 was absent mainly in the cerebral cortex (Ctx) and hippocampus (Hip) of the *Rack7*‐cKO mice, but not other brain regions such as the thalamus (Th) (Figure S2b, Supporting Information).

**Figure 2:**
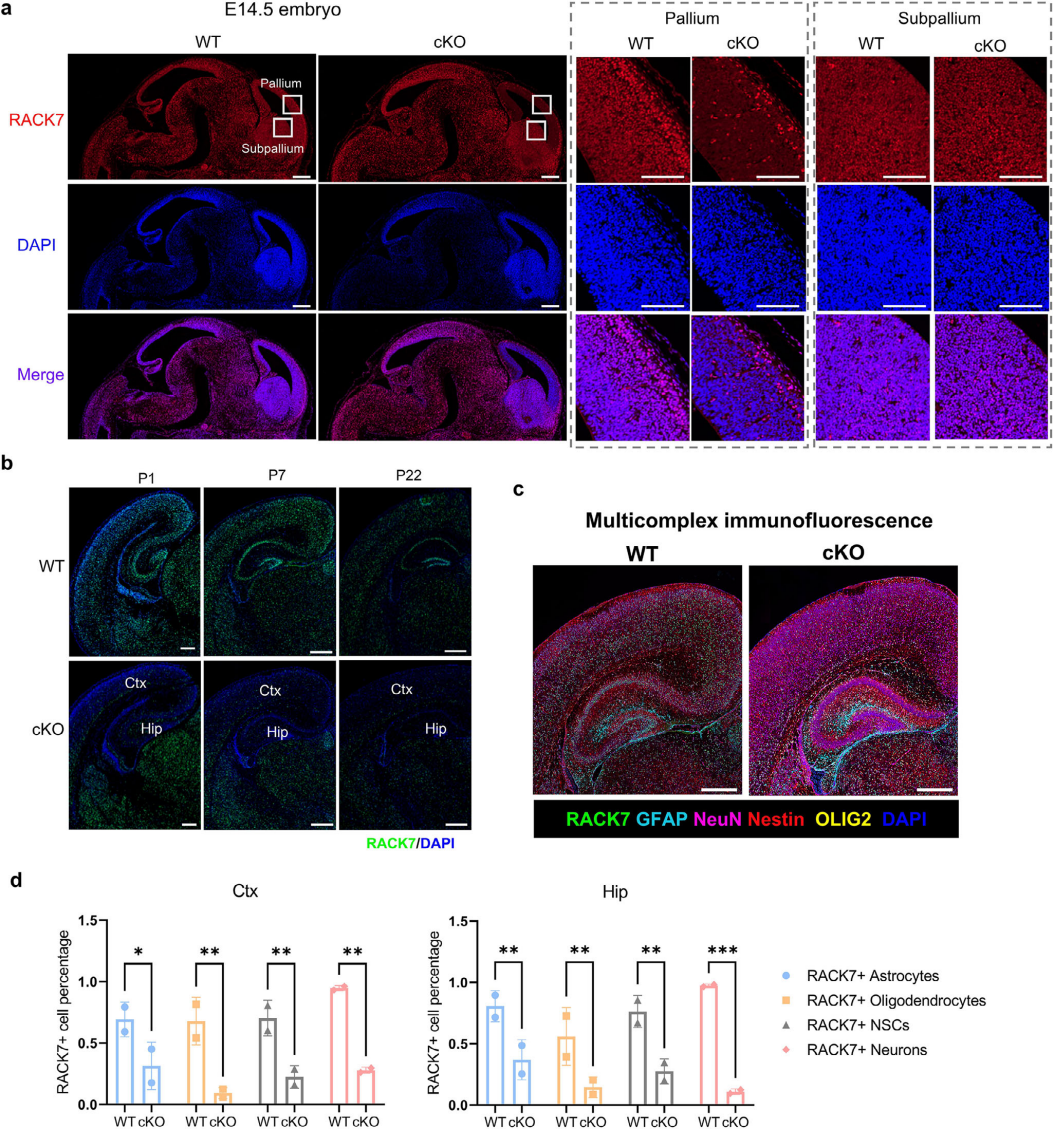
RACK7 deletion is primarily observed in the cerebral cortex and hippocampus. a) IF images against RACK7 in WT and *Rack7*‐cKO embryonic sections show RACK7 specifically deletion in the pallium of forebrain as early as E14.5. Scale bars = 200 µm. b) IF staining images of RACK7 in WT and *Rack7*‐cKO brain slides at P1, P7 and P22. Scale bars = 400 µm. c) mIF images in WT and *Rack7*‐cKO brain slides at P7. Antibodies against RACK7, NeuN, Nestin, GFAP, OLIG2 and DAPI. Scale bars = 400 µm. d) Quantitative analysis of the proportion of RACK7+ cells among various cell types in Ctx and Hip of WT and *Rack7*‐cKO brain slides at P7. Cell types in the brain were multicomplex IF stained with cell‐specific markers as shown in c). Data are presented as the mean ± SD, statistical significance was determined using two‐way ANOVA; *p ≤ 0.05, **p ≤ 0.01, ***p ≤ 0.001.

The hGFAP‐Cre promoter was activated as early as E13.5,^[^ ^17^ ^]^ while the developmental phenotypes caused by *Rack7* deletion appeared after birth. So, we first examined RACK7 deletion using immunofluorescence (IF) in E14.5 embryos. We found that RACK7 protein was predominantly absent in the pallium and pallial ventricular zones (VZs) of the *Rack7*‐cKO forebrain, which develops into the cerebral cortex and hippocampus postnatally. In contrast, no specific loss of RACK7 was detected in the subpallium, consisting with previous report^[^ ^18^ ^]^ (**Figure** **2**a).

We then performed IF staining for RACK7 in brain tissue sections isolated from *Rack7*‐cKO mice and their WT littermates at neonatal (P1), P7, and P21, which are crucial for postnatal neuronal and astrocyte development.^[^ ^19^ ^]^ The IF staining and subsequent analysis of *Rack7*‐cKO mice compared to wildtype controls revealed that the loss of RACK7 is predominantly observed in the Ctx and Hip regions across postnatal brain development (Figure 2b; Figure S2c, Supporting Information).

To investigate the loss of RACK7 protein across various brain cell types, we performed multicomplex immunofluorescence (mIF) staining for RACK7, NeuN (a marker for neurons), Nestin (a marker for neural stem cells, NSCs), GFAP (a marker for astrocytes), OLIG2 (a marker for oligodendrocytes) and DAPI (to stain all nuclei) in brain tissue sections from *Rack7‐*cKO mice and their WT littermates at P7 (Figure 2c; Figure S2d,e, Supporting Information). For the quantitative analysis of the mIF results, we selected two regions of interest (ROIs), including Ctx and Hip (Figure S2f, Supporting Information). In *Rack7*‐cKO mice, we consistently observed a loss of RACK7 in the Ctx and Hip (Figure S2g, Supporting Information). Notably, quantitative analysis revealed that RACK7 deletion occurred across different cell types in the Ctx and Hip (Figure 2d; Figure S3, Supporting Information).

These results demonstrate the specific loss of RACK7 protein in the cortical and hippocampal region, from the initial activation of Cre during embryonic stages to the period when the developmental defects manifestation.

### 2.3 Transcriptional Landscape Upon Rack7 Deletion at Embryonic Day 14.5

RACK7 has been identified as a transcriptional repressor, to obtain more precise insights into the consequences of RACK7 loss, we utilized high‐throughput single‐cell RNA sequencing to examine the transcriptional landscapes of *Rack7*‐cKO and wildtype brains. We first dissected E14.5 forebrain tissues from 3 WT and 3 *Rack7*‐cKO embryos and dissociated the tissues into single cells prior to processing them on the 10x Chromium platform (10x Genomics). After removing low‐quality cells and doublets, 10186 cells from WT and 8880 cells from *Rack7*‐cKO sample remained, respectively. We then combined cells from WT and *Rack7*‐cKO embryos for clustering analysis and obtained 24 distinct cell clusters (Figure S4a, Supporting Information). Based on the expression of known marker genes,^[^ ^20^, ^21^ ^]^ we annotated the major cell‐type in the pallium and pallial VZs of the forebrain (Figure S4b,c, Supporting Information), including the forebrain excitatory neuron (Ex_neuron) (*Neurod6*+),^[^ ^22^ ^]^ radial glial cell (RGC) (*Pax6*+), and neuronal intermediate progenitor (IP) (*Eomes*+). We further analyzed the cell numbers but observed no significant alteration in cell proportions upon *Rack*7 deletion (Figure S4d,e, Supporting Information).

As RACK7 protein is predominantly deleted in the pallium of forebrain (Figure 2a), we then compared transcriptional profiles within each of the main cortical cell types (Ex_neuron, IP, and RGC) between WT and *Rack7*‐cKO groups (with cutoff log_2_(Fold Change (FC)) > 0.25 and *p‐adjust* < 0.05). As a result, the total number of differentially expressed genes (DEGs) was found to be minimal (Figure S4f, Supporting Information). These results suggest that, despite the early expression of hGFAP‐Cre at E13.5 and the confirmed deletion of RACK7 in the *Rack7*‐cKO forebrain, RACK7 does not exert significant transcriptional repression at embryonic day E14.5.

### 2.4 Cell Type Transcriptional Changes in *Rack7*‐cKO Cerebral Cortex and Hippocampus

Given that the phenotype of *Rack7*‐cKO mice gradually intensifies postnatally, we aimed to investigate the transcriptional repression effects in postnatal mice. So, we extracted the cortical and hippocampal tissues where RACK7 protein is predominantly deleted (Figure 2a,b), from *Rack7*‐cKO and WT mice at P3 and P22 respectively, prior to processing with the 10X Genomics platform.

**Figure 3:**
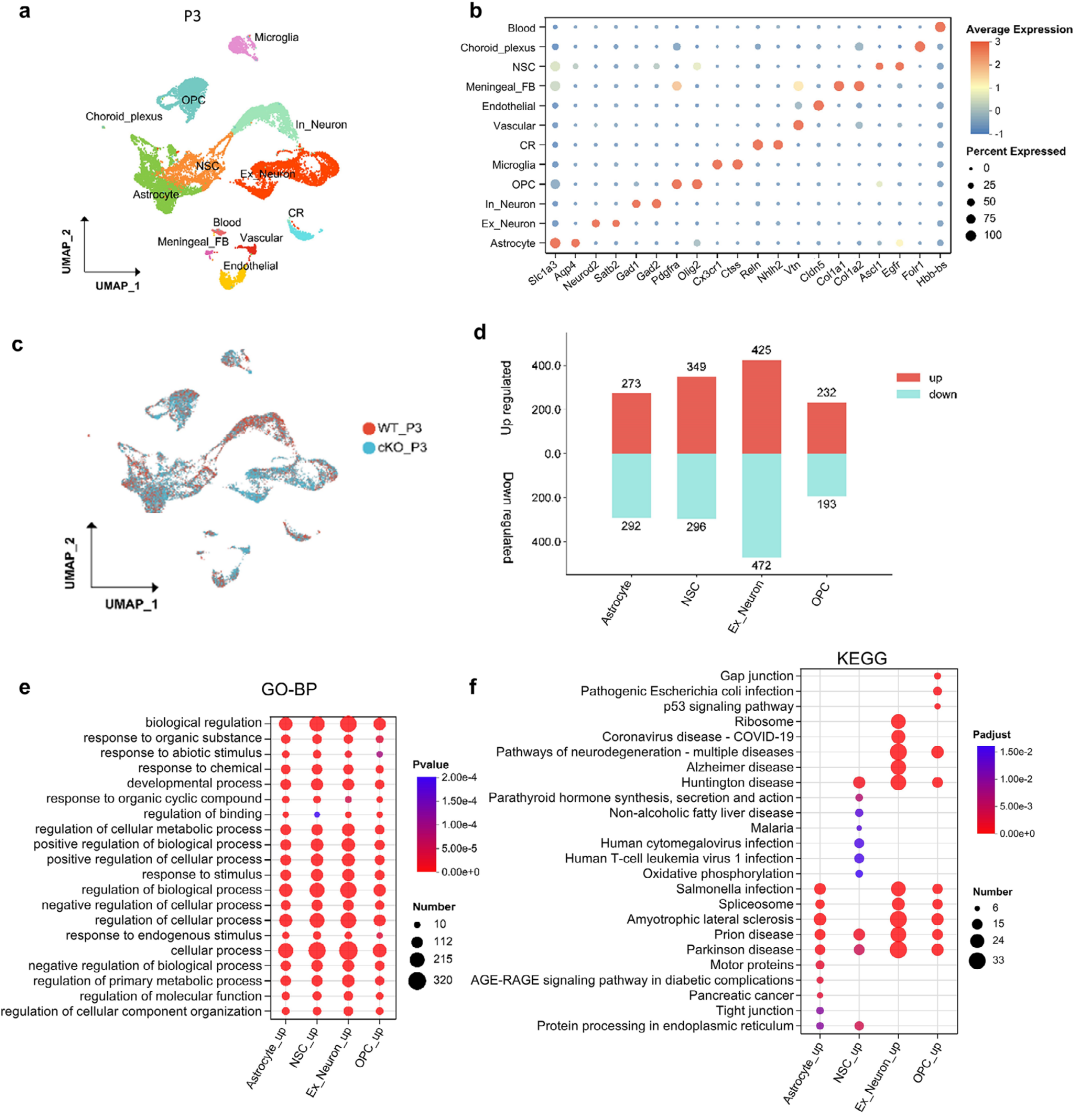
Cell type–specific transcriptional changes in the cerebral cortex and hippocampus of P3 *Rack7*‐cKO and control mice. a) Uniform manifold approximation and projection (UMAP) plot showing the major cell types. In which, cell types are labeled as Ex_neuron, excitatory neuron; In_neuron, inhibitory neuron; CR, Cajal‐Retzius cell; OPC, oligodendrocyte precursor cell; NSC, postnatal neural stem cell; Choroid_plexus, choroid plexus epithelial cell; Meningeal_FB, meningeal fibroblast. b) Dot plot showing the expression of selective markers in different cell types. c) UMAP plot showing the cells from different genotypes, which were labeled with different colors. d) Bar graph showing the number of DEGs in indicated cell populations. e) GO analysis of up‐regulated genes from indicated cell populations. Top10 GO terms from BP were ordered by *p*‐value. f) KEGG pathway analysis of up‐regulated genes from indicated cell populations. Top10 KEGG terms were ordered by *p‐adjust*.

For P3 mice tissues, after removing low‐quality cells and doublets, our initial comparison of the transcriptomes from 6512 cells of wild‐type and 11188 cells of *Rack7*‐cKO mice revealed 30 distinct cell clusters (Figure S5a, Supporting Information). Based on their unique expression profiles, we annotated these clusters into three types of neurons, including excitatory neuron (Ex_neuron) (*Neurod2*+), inhibitory neuron (In_neuron) (*Gad2*+) and Cajal‐Retzius cell (CR) (*Nhlh2*+), two types of glial cells, including oligodendrocyte precursor cell (OPC) (*Pdgfra+*) and astrocyte (*Slc1a*3+), a proportion of postnatal neural stem cell (NSC) (*Ascl1*+ and *Egfr*+), microglia (*Cx3cr1*+) and other cell types, i.e., choroid plexus epithelial cell (Choroid_plexus) (*Folr*1+ and *Prlr*+), endothelial cell (Endothelial) (*Cldn5*+), meningeal fibroblast (Meningeal_FB) (*Col1a1*+ and *Col1a2*+), vascular cell (Vascular) (*Vtn*+), and blood cell (Blood) (*Hbb‐bs*+) (**Figure** **3**a,b; Figure S5b, Supporting Information).^[^ ^23^, ^24^, ^25^, ^26^, ^27^ ^]^ By statistically analyzed the cell proportion within each sample, we observed an increased proportion of excitatory neurons (13.53% to 24.03%) and a decreased proportion of inhibitory neurons (20.95% to 8.64%) (Figure 3c; Figure S5c, Supporting Information), however, no new cell sub‐populations were identified within these two cell types (Figure S5d–g, Supporting Information).

To identify the changes in gene expression induced by RACK7 loss in a cell type‐specific context, we compared transcriptional profiles of each cell type derived from pallium and pallial VZs, compromising the astrocyte, Ex_Neuron, NSC, and OPC,^[^ ^18^, ^20^, ^21^, ^28^ ^]^ between the WT and *Rack7*‐cKO mice (with cut‐off *p*‐value < 0.05, log2(FC) ≥ 0.25) (Figure 3d). Our results indicated that the loss of RACK7 led to transcriptional changes across cell types developed from pallium and pallial VZs.

To elucidate the functional implications of these transcriptional changes, we conducted Gene Ontology (GO) and Kyoto Encyclopedia of Genes and Genomes (KEGG) pathway analyses on the up‐regulated genes and highlighting the top 10 terms from each of the five cell types (Figure 3e; Figure S6, Supporting Information). Biological process (BP) of GO analysis suggests that these genes are enriched in terms related to cellular process (GO:0 009987) and developmental process (GO:00 32502). Additionally, we identified the terms regulation of cellular metabolic process and regulation of primary metabolic process (GO:00 31323, GO:00 80090) in Ex_Neuron, a process that cooperated by astrocytes and contributes to behavior and brain diseases^[^ ^29^ ^]^ as well as the response to stimuli (GO:00 50896) in NSC, OPC and astrocyte, which reflects the activation of cell‐cell interactions among these cells or with other cells (Figure S6, Supporting Information). Notably, terms related to Parkinson's disease, Prion disease, and Huntington's disease were enriched across these cell types (Figure 3f), suggesting that dysregulation of RACK7 could be implicated in several CNS diseases.

**Figure 4:**
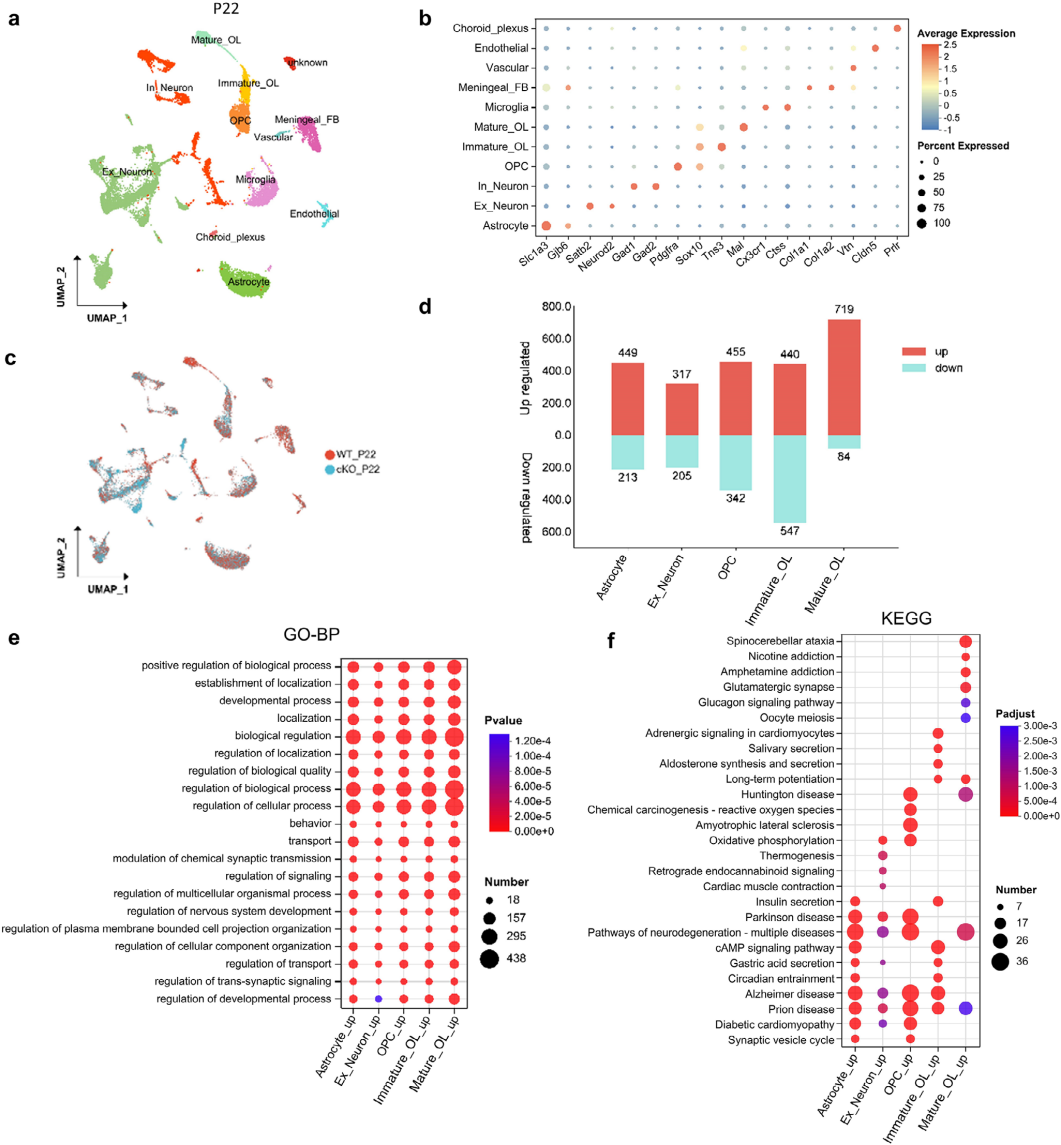
Cell type–specific transcriptional changes in the cerebral cortex and hippocampus of P22 *Rack7*‐cKO and control mice. a) UMAP plot showing the major cell types. In which, cell types are labeled as Ex_neuron, excitatory neuron; In_neuron, inhibitory neuron; OPC, oligodendrocyte precursor cell; Choroid_plexus, choroid plexus epithelial cell; Meningeal_FB, meningeal fibroblast; Immature_OL, immature oligodendrocyte and Mature_OL, mature oligodendrocyte. b) Dot plot showing the expression of selective markers in different cell types. c) UMAP plot showing the cells from different genotypes, which were labeled with different colors. d) Bar graph showing the number of DEGs in indicated cell populations. e) GO analysis of up‐regulated genes from indicated cell populations. Top10 GO terms from BP were ordered by *p*‐value. f) KEGG pathway analysis of up‐regulated genes from indicated cell populations. Top10 KEGG terms were ordered by *p‐adjust*.

Given the increased severity of behavioral abnormalities and substantial mortality in *Rack7*‐cKO mice between 15 to 30 days postnatal (Figure 1b,e–h), we also performed single‐nucleus transcriptomic analysis (snRNA‐seq) on cortical and hippocampal tissues from *Rack7*‐cKO and wild‐type mice at P22. We compared the transcriptomes of 11156 cells from wild‐type and 10327 cells from *Rack7*‐cKO mice at P22, identifying 34 cell clusters (Figure S7a, Supporting Information). These clusters were further annotated as two types of neurons, including excitatory neuron (Ex_neuron)(*Satb2*+) and inhibitory neuron (In_neuron)(*Gad2*+), three lineages of oligodendrocytes, including oligodendrocyte precursor cell (OPC)(*Pdgfra*+), immature oligodendrocyte (Immature_OL)(*Tns3*+) and mature oligodendrocyte (Mature_OL)(*Mal*+), astrocyte(*Gjb6*+), microglia(*Cx3xr1*+), as well as cell types, i.e., choroid plexus epithelial cell (Choroid_plexus) (*Folr*1+ and *Prlr*+), endothelial cell (Endothelial) (*Cldn5*+), meningeal fibroblast (Meningeal_FB) (*Col1a1*+ and *Col1a2*+) and vascular cell (Vascular)(*Vtn*+) (**Figure** **4**a,b; Figure S7b, Supporting Information).^[^ ^25^, ^26^, ^27^ ^]^ We also observed an increased proportion of excitatory neurons in *Rack7*‐cKO mice, rising from 22.36% to 45.35% (Figure S7c, Supporting Information) as shown in P3. Additionally, no new cell sub‐populations were detected (Figure 4c; Figure S7d,e, Supporting Information). Unlike in P3, we did not find any alteration in the proportion of the inhibitory neurons cluster between *Rack7*‐cKO and wild‐type mice at P22.

The transcriptional profiles exhibited DEGs in astrocytes, excitatory neurons, immature, and mature oligodendrocytes (with cut‐off *p*‐value < 0.05, log2(FC) ≥ 0.25) (Figure 4d). In the P22 dataset, RACK7 displayed a more pronounced transcriptional repression activity, with a higher number of upregulated genes compared to downregulated genes across several cell types. The top10 GO and KEGG terms of up‐regulated genes in each cell type revealed enrichment for developmental processes as well as CNS diseases, including Alzheimer's disease, prion disease, and Parkinson's disease (Figure 4e,f). In contract, the top10 KEGG terms for down‐regulated genes in each cell type exhibited lower significant and did not show a clear association with CNS diseases (Figure S7f, Supporting Information).

Particularly, in the GO analysis in different cell types at P22 (Figure 4e; Figure S8, Supporting Information), we found the regulation of transport (GO:00 51049) regulated by RACK7 in glial cells, which reflects the activation of cell‐cell interactions among these cells or with other cells. We also identified behavior (GO:0 007610) notably in Ex_neuron as well as other cell types, which is associated with behavioral alterations during this stage.

In aggregate, our single cell/nucleus RNA‐seq results from E14.5, P3, and P22 *Rack7*‐cKO mice demonstrate that the loss of RACK7 doesn't affect gene transcription at early neurogenesis stage (E14.5), it contributes to the dysregulation of genes associated with developmental process and CNS diseases across several cell types (P3 and P22).

### 2.5 *Rack7*‐cKO Astrocyte Show Altered Transcription Profiles

Astrocytes, the most abundant type of glial cell, play pivotal roles in providing structural support, supplying nutrients, modulating synapses, and facilitating neuronal development. While numerous studies on neurodevelopmental diseases have focused on intrinsic changes within neurons, emerging work has demonstrated that glial cells also play a crucial role.^[^ ^30^, ^31^ ^]^ Cortical astrocytes are generated from RGCs around the time of birth, with their production and maturation continuing until the third postnatal week (P21).^[^ ^23^ ^]^ Notably, RACK7 deletion driven by hGFAP‐cre occurred at E13.5 in the pallium and pallial VZs (Figure 2a), with the developmental defects induced by RACK7 deletion mainly appeared around P14 (Figure 1b–e). This timing suggests that the loss of RACK7 in astrocytes may play a role in these processes. Furthermore, functional analysis of single cell/nucleus RNA‐seq data from P3 and P22 revealed that the enriched GO terms were related to cell‐cell communication and neuron metabolism (Figures S6 and S8, Supporting Information), which involve astrocytes.

**Figure 5:**
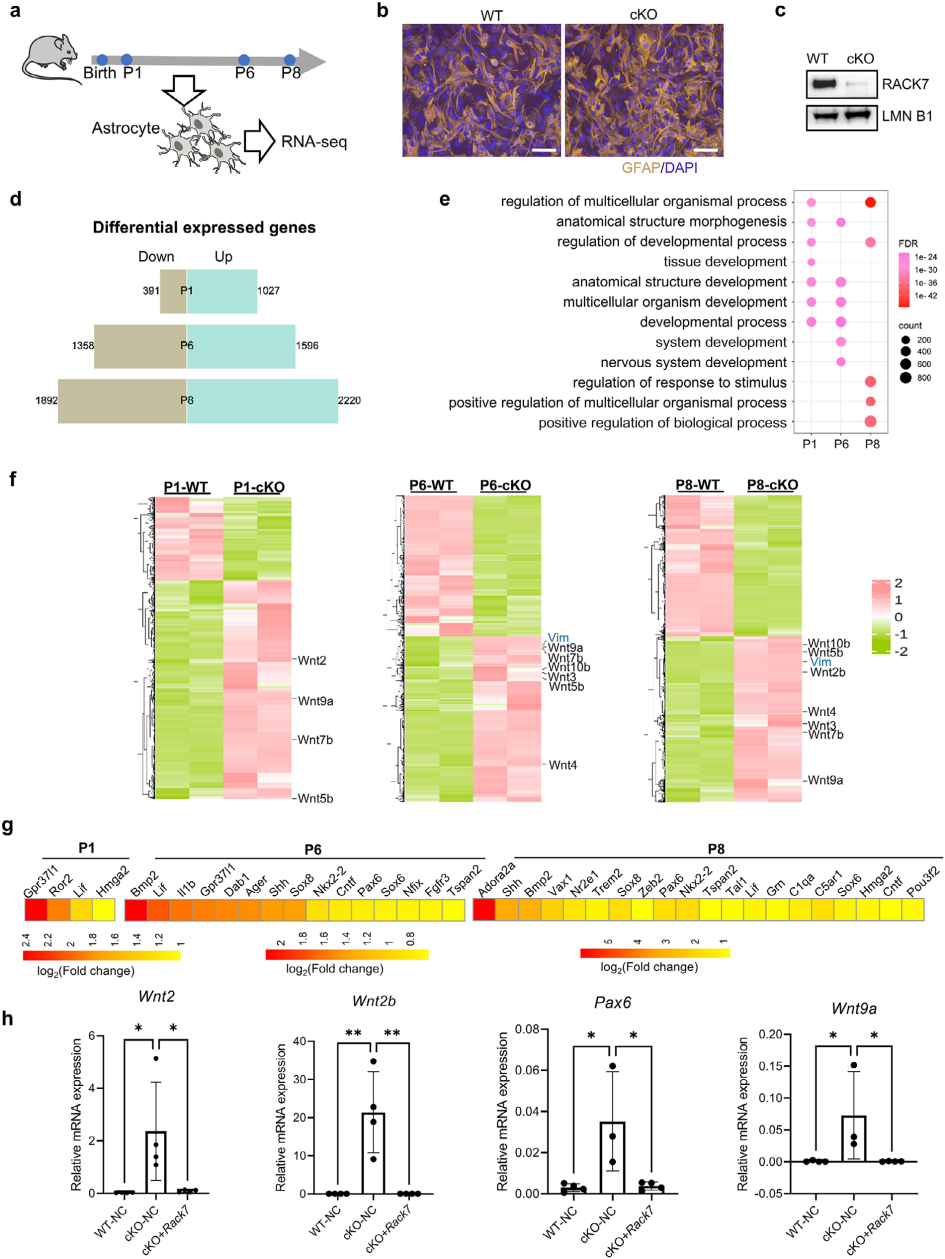
*Rack7*‐cKO astrocytes show altered transcription profiles. a) Schematic representation of the strategy for transcriptome profiling. Primary astrocytes were extracted and cultured from the brains of WT and *Rack7*‐cKO mice at neonatal, P6, and P8, followed by RNA‐seq analysis. b) IF images of GFAP in WT and *Rack7*‐cKO primary astrocytes. c) Immunoblots of RACK7 in WT and *Rack7*‐cKO primary astrocytes. d) DEGs between WT and *Rack7*‐cKO primary astrocytes (FC >1.5, *p*‐value < 0.05) at P1, P6 and P8. e) GO analysis of up‐regulated genes from d). GO terms from BP were ordered by *FDR*. f) Heatmap of differentially expressed genes shown in d). Wnt genes were up‐regulated in *Rack7*‐cKO primary astrocytes at P1, P6, and P8. g) Genes associated with astrocyte development (GO:00 14002) and astrocyte differentiation (GO:00 48708) show increased expression in *Rack7*‐cKO primary astrocytes at P1, P6 and P8, respectively. h) Relative mRNA expression of indicated genes in WT, *Rack7*‐cKO, and *Rack7*‐cKO primary astrocytes rescued with exogenous *Rack7* transgene. Data are presented as the mean ± SD, statistical significance was determined using one‐way ANOVA; *p ≤ 0.05, **p ≤ 0.01.

For these reasons, we purified primary astrocytes to further investigate how RACK7 regulates gene transcription through chromatin binding. In rodents, cortical astrocyte generation begins at E18/P0 and peaks at about P8,^[^ ^32^ ^]^ so we extracted and cultured primary astrocytes from WT and *Rack7*‐cKO mice brains at three‐time points, including neonatal (P1), P6 and P8, and performed RNA‐seq analysis (**Figure** **5**a). The identity of astrocytes and RACK7 protein level in WT and *Rack7*‐cKO primary astrocytes were further confirmed by immunostaining of the astroglial marker GFAP (Figure 5b) and immunoblotting of RACK7 (Figure 5c), respectively. RNA‐seq identified an increasing number of differentially expressed genes during postnatal development stages of P1 (up: 1027, down: 391), P6 (up: 1596, down: 1358), and P8 (up: 2220, down: 1892) when *Rack7*‐cKO astrocytes were compared with WT (with cut‐off FC >1.5 and *p*‐value < 0.05). The repressive impact of *Rack7* on gene transcription appeared to be amplified during postnatal astrocyte development (Figure 5d). To explore the biological significance of the up‐regulated genes in *Rack7*‐cKO astrocytes, we performed GO analysis. We found enrichment of similar GO terms across the different postnatal days, including regulation of multicellular organismal process and developmental process (Figure 5e), consistent with the results from sc/sn RNA‐seq at P3 and P22 (Figures 3e and 4e).

Similarly, we also analyzed differentially expressed genes using a 9‐square plot that classified genes into nine groups according to the log_2_(FC) at P6 versus P1 and P8 versus P6 in both WT (x‐axis) and *Rack7*‐cKO astrocyte cells (y‐axis) (Figure S9a, Supporting Information). We identified genes with decreased expression in WT but increased expression in cKO (group A), and increased expression in WT but decreased expression in cKO (group I) (with cut‐off 1.5‐FC and *p*‐value < 0.05) (Figure S9b, Supporting Information). GO analysis of these genes (group A+D, group F+I) showed similar GO terms related to developmental process (Figure S9c, Supporting Information).

Surprisingly, we found multiple Wnt ligands (Wnts) up‐regulated in our RNA‐seq data (P1: Wnts = 4; P6: Wnts = 6; P8: Wnts = 7), such as *Wnt5b, Wnt7b*, and *Wnt9a*, which are up‐regulated in neonatal, P6 and P8 (Figure 5f). Wnt signaling pathways play a crucial role throughout all stages of brain development and whose defects are linked to many neurological disorders.^[^ ^33^, ^34^ ^]^

Furthermore, in addition to Wnt genes, other genes related to astrocyte development (GO:00 14002) and astrocyte differentiation (GO:00 48708) have also been found to show an increased expression in response to RACK7 loss (Figure 5g). These genes are known to be important for the activation of astrocyte differentiation during central nervous development, such as the *Sox* genes family transcription factors *Sox6* and *Sox8*, astrocyte‐specific G protein‐coupled receptor *Gpr37l1*, bone morphogenetic protein (BMP) signaling gene *Bmp2*, and Sonic hedgehog (Shh) signaling gene *Shh*.^[^ ^35^, ^36^, ^37^, ^38^, ^39^ ^]^ Notably, we introduced an exogenous *Rack7* transgene into *Rack7*‐cKO primary astrocytes and found that the elevated expression of Wnt and astrocyte developmental genes upon *Rack7* deletion was decreased (Figure 5h). This finding confirms that the transcriptional alterations were indeed caused by *Rack7* deficency. Collectively, these findings indicate *Rack7* loss in primary astrocytes impacts astrocyte development at the transcription level with upregulation of Wnt genes.

### 2.6 Molecular Mechanisms of *Rack7*‐Mediated Gene Regulation

**Figure 6:**
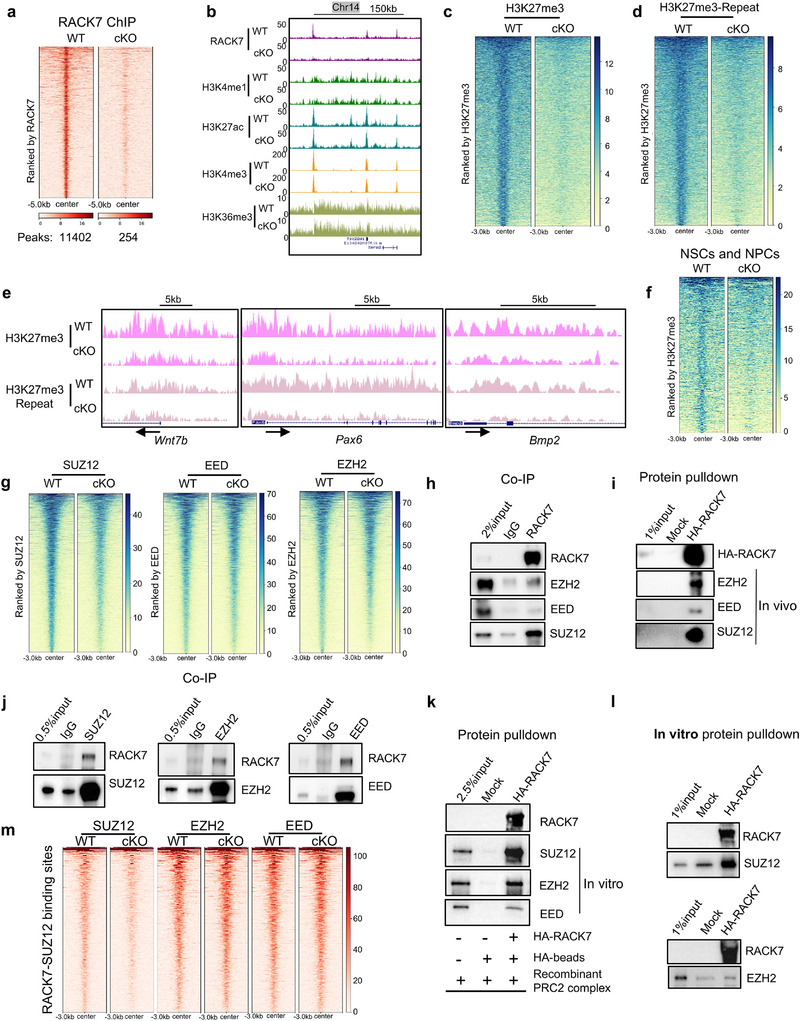
Molecular mechanisms of *Rack7*‐mediated gene regulation. a) Heatmap analysis of RACK7 ChIP‐seq signals in WT and *Rack7*‐cKO primary astrocytes. b) UCSC genome browser snapshot of indicated ChIP‐seq signals in WT and *Rack7*‐cKO primary astrocytes. c) Heatmap analysis of H3K27me3 ChIP‐seq signals in WT and *Rack7*‐cKO primary astrocytes extracted from P1 mice forebrain. d) Biologically replicate of H3K27me3 ChIP‐seq signals in WT and *Rack7*‐cKO astrocytes at P6. e) UCSC Genome browser snapshots of H3K27me3 ChIP‐seq signals in WT and *Rack7*‐cKO astrocytes, data from two biological replicates. f) Heatmap analysis of H3K27me3 ChIP‐seq signals in WT and *Rack7*‐cKO primary neural stem and progenitor cells (NSCs and NPCs). g) Heatmap analysis of ChIP‐seq signals of PRC2 complex components, SUZ12, EED and EZH2 in WT and *Rack7*‐cKO astrocytes. h) Co‐IP analysis using an anti‐RACK7 antibody demonstrating the interaction between RACK7 and core PRC2 complex in WT neonatal forebrain. i) Protein pulldown analysis utilizing an exogenous HA‐tagged full‐length human RACK7 protein in WT neonatal forebrain nuclear extract. j) Co‐IP analysis using anti‐SUZ12, EZH2, EED antibodies demonstrating the interaction between RACK7 and core PRC2 complex in WT neonatal forebrain. k) Protein pulldown analysis utilizing recombinant HA‐tagged full‐length human RACK7 protein and recombinant PRC2 complex protein (Active Motif). l) Protein pulldown analysis utilizing recombinant HA‐tagged full‐length human RACK7 protein and recombinant EZH2 or SUZ12. m) Heatmap analysis of SUZ12, EZH2 and EED ChIP‐seq signals at RACK7‐SUZ12 co‐binding sites.

To gain further insight into the molecular mechanisms underlying *Rack7* function, we first performed chromatin immunoprecipitation coupled with deep sequencing (ChIP‐seq) using an anti‐RACK7 antibody to determine the genomic locations of RACK7 in primary astrocytes. Our ChIP‐seq analysis identified 11402 RACK7 peaks in WT astrocytes as opposed to 254 RACK7 peaks in the *Rack7*‐cKO astrocytes (**Figure** **6**a,b), indicating most if not all the peaks detected in the wildtype astrocytes are bona fide RACK7 binding events.

RACK7 has previously been shown to bind multiple histone modifications.^[^ ^7^, ^11^ ^]^ To investigate whether RACK7 knockout affects the distribution of histone modifications, we performed ChIP‐seq analysis on various histone modifications, compromising H3K4me1, H3K27ac, H3K4me3, H3K36me3 and H3K27me3 (Figure 6b; Figure S10a, Supporting Information). Surprisingly, we observed a significant decrease in the chromatin enrichment but not the global protein expression of histone H3K27me3 in *Rack7*‐cKO astrocytes, while no notable global changes in other histone modifications both in chromatin occupancy and protein level (Figure 6b,c; Figure S10a–e, Supporting Information). To rule out potential experimental bias, we performed additional biological replicates using primary astrocytes at P6 and consistently observed a reduction in H3K27me3 chromatin enrichment in the *Rack7*‐cKO cells (Figure 6d,e).

We next sought to determine whether the alteration of H3K27me3 is cell‐type specific and restricted to primary astrocytes. To address this, we purified and cultured neural stem and progenitor cells (NSC and NPCs) both from WT and *Rack7*‐cKO mice brains. Immunoblot analysis showed selective RACK7 protein loss in the *Rack7*‐cKO NSC and NPCs (Figure S10f, Supporting Information). Consistently, we found decreased chromatin distributions of H3K27me3 in *Rack7*‐cKO NSC and NPCs (Figure 6f). These data suggest RACK7 plays a regulatory role in H3K27me3 chromatin distribution across CNS cell types.

H3K27me3 plays a crucial role during mammalian embryonic development by silencing the expression of key developmental genes.^[^ ^40^ ^]^ In mammals, H3K27me3 is catalyzed by PRC2 complex, which contains a trimeric core of the enhancer of EZH2, embryonic ectoderm development (EED) and SUZ12.^[^ ^41^, ^42^ ^]^ PRC2 exists in two distinct forms, PRC2.1 and PRC2.2, which are defined by their specific accessory proteins: PRC2.1 (PCL1–3, EPOP, and PALI1/2) and PRC2.2 (AEBP2 and JARID2).^[^ ^43^ ^]^ SUZ12 serves as a scaffold, facilitating interactions with EZH2 and EED, as well as with the subunits RBBP4/7, and accessory proteins, thereby contributing to the establishment of the H3K27me3 modification.^[^ ^44^ ^]^ The knockout of these accessory proteins such as PCL1‐3, JARID2 could impact the distribution of SUZ12 and H3K27me3, but it does not affect the global protein level of H3K27me3,^[^ ^43^, ^45^ ^]^ which is consistent with our results, suggesting that RACK7 may have similar functions.

Given that we didn't observe significant changes both in the transcription and protein levels of the PRC2 components (Figure S10g–i, Supporting Information), we subsequently analyzed the genomic occupancy of three PRC2 core components, EZH2, EED, and SUZ12 by ChIP‐seq in both WT and *Rack7*‐cKO astrocytes. Global analysis of ChIP‐seq peaks showed decreased genomic occupancy of all three PRC2 core components, especially for the SUZ12 (Figure 6g).

To investigate whether RACK7 interacts with the PRC2 complex to regulate genomic occupancy of the PRC2 complex and histone H3K27me3 modification, we performed co‐immunoprecipitation (Co‐IP) in the nuclear extract from WT neonatal forebrain. Our data showed that RACK7 interacts with the core PRC2 components, including EZH2, EED, and SUZ12, in vivo (Figure 6h). We further confirmed these interactions with an Hemagglutinin (HA)‐tagged full‐length RACK7 protein in the nuclear extracts from WT neonatal forebrain (Figure 6i). Furthermore, we validated that RACK7 could be co‐immunoprecipitated by antibodies against EZH2, EED, and SUZ12 (Figure 6j).

To further clarify the interaction between RACK7 and PRC2 complex, we conducted an in vitro binding assay using recombinant RACK7 and the PRC2 complex (Figure 6k). The results demonstrated that RACK7 can bind to the PRC2 complex in vitro. Based on that, we performed additional in vitro binding assays with recombinant RACK7, EZH2, or SUZ12, revealing that RACK7 preferentially interacts with SUZ12 (Figure 6l). Moreover, in the genomic locations co‐occupied by RACK7 and SUZ12, we observed drastically reduced SUZ12 occupancy in the *Rack7*‐cKO astrocytes (Figure 6m).

Our findings indicate that RACK7 interactions with core PRC2 complex, specifically the SUZ12, thereby helping to establish the genomic locations of the H3K27me3 modification.

### 2.7 RACK7 Works with H3K27me3 Modification to Suppress Gene Expression

**Figure 7:**
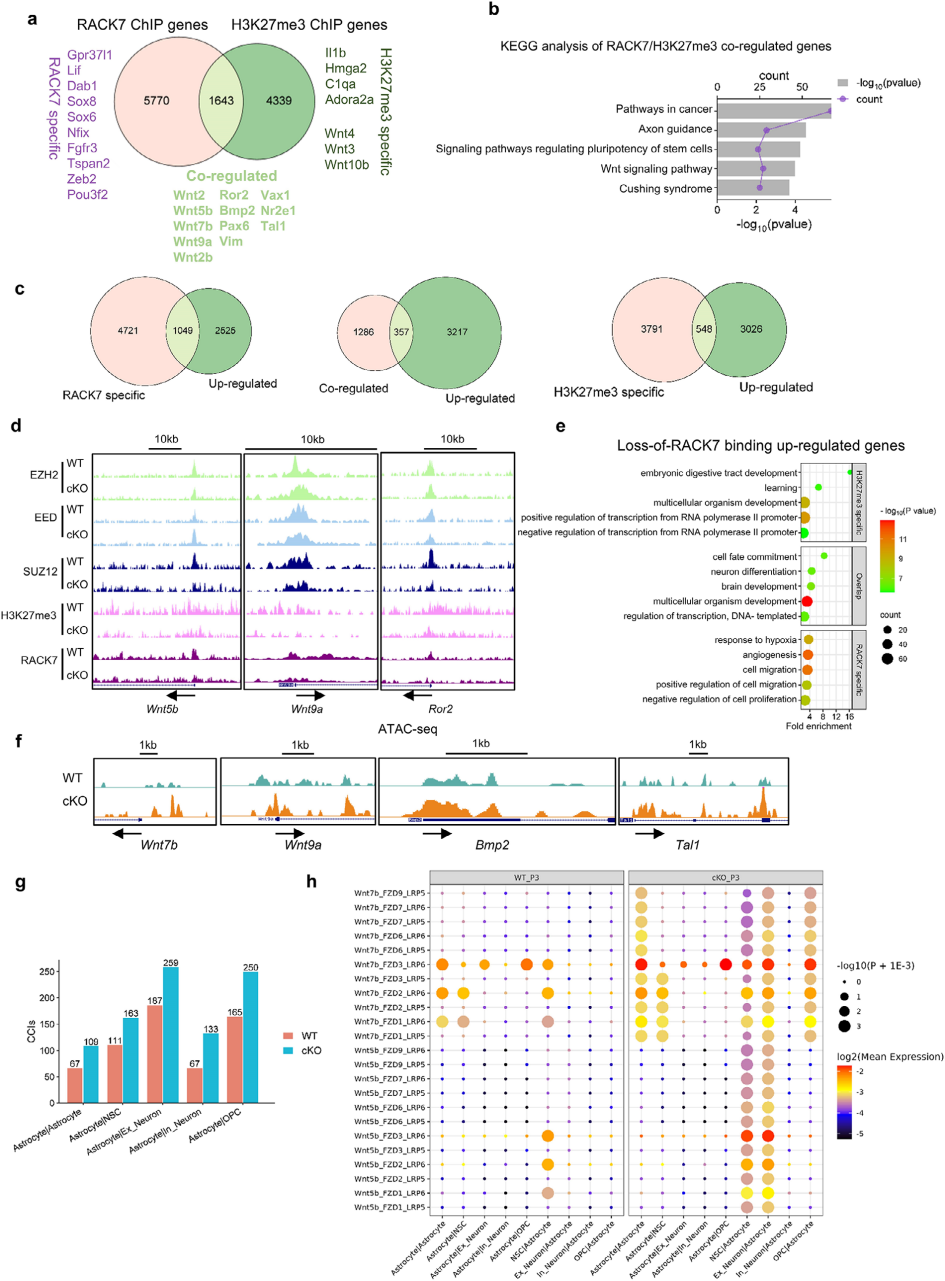
RACK7 works with H3K27me3 to repress transcription of genes related to brain development. a) Venn diagram analysis of RACK7 and H3K27me3 ChIP‐seq peak‐annotated genes in WT primary astrocytes. Wnt genes and genes involved in astrocyte development and differentiation are highlighted. b) KEGG analysis of 1643 RACK7/H3K27me3 co‐regulated genes. c) Venn diagram illustrating all up‐regulated genes from Figure 5d and three categories of ChIP‐seq targets from a). d) UCSC Genome browser snapshots of selected ChIP‐seq signals in WT and *Rack7*‐cKO astrocytes. Wnt genes and astrocytic genes co‐regulated by RACK7 and H3K27me3 are shown. e) GO analysis of 1049, 548, and 357 genes from the three comparisons in c). f) UCSC Genome browser snapshots of selected ATAC‐seq signals in WT and *Rack7*‐cKO astrocytes. g) Total count of CCIs between astrocytes and other nervous cell clusters analyzed by CellphoneDB using P3 scRNA‐seq dataset. h) CCIs involving *Wnt7b* and *Wnt5b* with their receptors in WT and *Rack7*‐cKO brain at P3.

As discussed above, we found an elevation of transcription of Wnt genes in primary astrocytes when *Rack7* is deleted. Given that both RACK7 and H3K27me3 play a transcriptional repressive role, we therefore sought to determine whether RACK7 and H3K27me3 played a role in this regulation. We performed Venn analysis of the RACK7 and H3K27me3 ChIP‐seq peak annotated genes, identifying RACK7‐specific (*n* = 5770), H3K27me3‐specific (*n* = 4339) and RACK7/H3K27me3 co‐regulated genes (*n* = 1643) (**Figure** **7**a). Strikingly, KEGG analysis of the 1643 RACK7/H3K27me3 co‐regulated genes revealed specific enrichment in the Wnt signaling pathway (Figure 7b; Figure S11a, Supporting Information).^[^ ^46^, ^47^ ^]^ Integrating all the up‐regulated genes (P1, P6, and P8 in Figure 6d) with each group of ChIP‐seq targets, we identified 1049, 548 and 357 up‐regulated genes fall into the “RACK7 specific”, “H3K27me3 specific” and “co‐regulated” categories, respectively (Figure 7c). Notably, most of the Wnt genes and astrocyte differentiation and developmental genes belong to this “co‐regulated” group, which suggests that combined action of RACK7 and H3K27me3 may play a role in their regulation (Figure 7d). Furthermore, GO pathway analysis of the three categories of up‐regulated genes also provided supporting evidence as only the genes from the “co‐regulated” group, but not “RACK7 specific” or “H3K27me3 specific” groups, show enrichment of genes in the pathways related to the observed phenotypes, such as brain development, neuron differentiation and cell fate commitment (Figure 7e). Consistently, Assay for Transposase‐Accessible Chromatin with high throughput sequencing (ATAC‐seq) in WT and *Rack7*‐cKO astrocytes found increased chromatin accessibility in the genes co‐regulated by RACK7 and H3K27me3 (Figure 7f). In contrast, the enrichment of other histone modifications such as H3K4me1, H3K4me3, H3K27ac, and H3K36me3 on Wnt and astrocyte developmental genes remained unchanged upon *Rack7* deletion (Figure S11b, Supporting Information). This finding confirms the predominant regulatory role of RACK7 and H3K27me3 on these genes.

The Wnt signaling pathway is activated when Wnt ligands bind to the Frizzled (FZD) receptor family and a member of the low‐density lipoprotein receptor‐related protein (LRP) family, LRP5 or LRP6, forming Wnt‐FZD‐LRP5/6 complexes that can be identified through scRNA‐seq data analysis.^[^ ^48^ ^]^ Our single‐cell transcriptional results from P3 and P22 also suggest that astrocytes may function through cell‐cell interactions (CCIs). Therefore, in order to investigate the CCIs between astrocytes communicated with other nervous cell clusters via Wnt signaling pathways, we applied CellPhoneDB,^[^ ^49^ ^]^ a computational approach that predicts CCIs by ligand‐receptor partners analysis, in P3 *Rack7*‐cKO compared to control mice. We observed a significant increase in CCIs counts between astrocytes and other cell types upon RACK7 deletion (Figure 7g). Notably, the interactions involving *Wnt7b* and *Wnt5b* with their respective receptor pairs were significantly elevated in *Rack7*‐cKO mice (Figure 7h). Collectively, these findings suggest that RACK7 may recruit PRC2 for H3K27 methylation to silence Wnt and other astrocyte developmental genes during astrocyte development, thus providing new insights into how astrocyte development regulated by Wnt, a process that remains poorly understood.

In contrast, our findings indicate that the morphology of astrocytes remains unchanged upon *Rack7* deletion, as demonstrated by IF staining and quantitative analysis^[^ ^50^ ^]^ of the astrocyte morphological markers GFAP and S100 calcium binding protein B (S100B) in Ctx and Hip at various developmental stages (Figure S12a–c, Supporting Information). Additionally, single‐cell transcriptional results from P3 and P22 further show that *Rack7* deletion does not lead to the emergence of specific astrocyte subtypes (Figure S12d, Supporting Information).

In summary, our data suggest that RACK7 represses transcription by recruiting PRC2 complex, impacting the genomic distribution of histone H3K27me3 modification. RACK7 and H3K27me3 work together to prevent over‐expression of Wnts and other developmental genes in purified astrocytes. Concurrently, DEGs resulting from RACK7 knockout are associated with developmental processes both in snRNA/scRNA‐seq and purified astrocytes. For this reason, deletion *Rack7* in mice brain leads to accumulating of developmental defects after birth and abnormal behaviors, such as growth retardation, motor coordination defects and anxiety‐related behaviors, and premature death. Our results provide new insight into the function of *Rack7* in brain development and disease.

## 3 Conclusion and Discussion

Epigenetic mechanisms play a crucial role in brain development and disease and this view is further supported by the discovery of *de novo* mutations identified on important epigenetic regulators in neurodevelopmental diseases. Emerging evidence suggests a role for the chromatin reader, RACK7, in CNS development, but most of these studies are limited to cell lines, and in vivo exploration and underly mechanisms remained unclear. This report provides for the first in vivo evidence for RACK7 in brain development and the underlying molecular mechanism, i.e., its ability to regulate histone H3K27me3 modification by impacting the genomic localizations of the PRC2 complex.

Our mouse model demonstrates that the knockout of *Rack7* at embryonic day E13.5 results in detectable RACK7 deletion as early as E14.5 in the pallium and pallial VZs of the forebrain (Figure 2a). However, scRNA‐seq reveals that only a limited number of genes are regulated by *Rack7* knockout. Postnatally, *Rack7*‐cKO mice exhibit RACK7 deletion in the Ctx and Hip, and they begin to show increasingly severe developmental phenotypes, including growth retardation, spontaneous seizures, motor coordination defects, anxiety‐related behaviors, and premature death (Figure 1). Furthermore, DEGs identified through sc/sn RNA‐seq of the forebrain at P3 and P21, as well as RNA‐seq of the primary astrocyte, indicate an association with developmental processes. In contrast, we deleted *Rack7* in adult mice using Nes‐cre/ERT2 (C57BL/6‐Tg (Nes‐cre/ERT2) KEisc/J) to achieve a homozygous conditional knockout of *Rack7* at about P30 (Tamoxifen injection from P21‐P28). However, these mice exhibited no significant changes in either body weight or survival rates (data not shown).

Mechanistically, our data show that RACK7 recruits the core PRC2 complex to impact SUZ12 chromatin localization and H3K27 trimethylation. This finding is somewhat surprising as previous proteomics studies didn't identify an interaction between RACK7 and the PRC2 components.^[^ ^51^ ^]^ Although another published study suggests an interaction between RACK7 and phosphorylated EZH2 in hypoxia‐exposed or von Hippel–Lindau (VHL)‐deficient clear cell renal cell carcinoma (ccRCC) cells, in which SUZ12 and EED were down‐regulated,^[^ ^52^ ^]^ no global alterations of H3K27me3 were detected in these cells.

In this study, we elucidate the roles of RACK7 in astrocyte development and its potential implications in neurodevelopmental diseases. As the published studies have shown,^[^ ^18^ ^]^ the cre activity of hGFAP‐Cre was detected in the dorsomedial area of the E13.5 cortices, which gives rise to forebrain excitory neurons. Consistent with this cre property, our results also demonstrated RACK7 deletion in the pallium and pallial VZs of E14.5 forebrain but not in the subpallium (Figure 2a). We further observed variations in the number of excitatory neurons at both postnatal day P3 and P22, along with a significant number of DEGs. Functional analysis of these DEGs revealed significant pathways regulated by RACK7, which cooperates with astrocytes and is related to behavior (Figures S6 and S8, Supporting Information). Notably, we detected an enhancement in cell‐cell communication, mediated by Wnt ligands and receptors, between astrocytes and other cell types (Figure 7h). Therefore, future research will focus on investigating the roles of RACK7 knockout in other neural cells throughout the developmental process.

In summary, we have identified a critical role for the chromatin reader RACK7 in astrocytes and brain development, via a mechanism that involves regulation of H3K27 methylation. Mutations of *RACK7* have been identified in multiple developmental/neurodevelopmental disorders, as compiled by The Human Gene Mutation Database (HGMD).^[^ ^53^ ^]^ Our findings provide new insights into chromatin regulation in CNS development and human diseases.

## 4 Experimental Section

### Generation of Conditional Knockout Rack7 Mice

Conditional knockout *Rack7* mice were generated by the Shanghai Model Organisms Center, Inc. as illustrated in Figure S1a (Supporting Information). Two guide RNAs (gRNAs) were designed using the CRISPOR tool(crispor.tefor.net) to target mouse *Rack7* exon 3 and 4 (Ensembl, ENSMUST00000109269.8). The sequences of the gRNAs were CACCCCGGTCAATTTGCCCT (gRNA1) and CACACAATTCCCACCCCCTC (gRNA2). Cas9 mRNA was obtained by in vitro transcription from the PX330 vector. The targeting vector was constructed using In‐Fusion cloning, with exons 3 and 4 of *Rack7* flanked by loxP sequence, so that they can be excised by Cre recombinase. The Cas9 mRNA, gRNAs, and targeting vector were injected into C57BL/6 zygotes. The genotype of the mice was determined by polymerase chain reaction (PCR) to identify homozygous flox mice. The primer sequences used were GCCACGCATTAAGCCACTCCTG (Primer P1) and CAAACAAAGAACTCGCACTGACTC (Primer P2).

The animal protocols (202011001S, 202310004S) were approved by the Animal Welfare Committee of Shanghai Medical College, Department of Laboratory Animal Science, Fudan University. Mice of both sexes were used for the experiments.

### ScRNA‐Seq and SnRNA‐Seq

ScRNA‐seq for E14.5 forebrain tissues was performed by Suzhou Dynamic Biosystems Co., Ltd (Suzhou, China), P3 and SnRNA‐seq for P22 cortical and hippocampal tissues were performed by Majorbio Biopharm Technology Co., Ltd (Shanghai, China) as below:

### Single Cell/Nuclei Suspension Preparation

For ScRNA‐seq, tissues were dissociated into single‐cell suspension with digestion solution comprising 0.25% Trypsin (Thermo Fisher, #25200‐072) and 10 µg mL^−1^ DNase I (Sigma, #11 284 932 001) with 5% Fetal Bovine Serum (FBS; Thermo Fisher, #SV30087.02) in PBS at 37 °C with a shaking speed of 50 rpm for ≈40 min. Cell suspensions were filtered through a 40 µm nylon cell strainer, and red blood cells were removed using Red Blood Cell Lysis Solution (Thermo Fisher, # 00‐4333‐57). The dissociated cells were washed with 1x DPBS buffer containing 0.4% FBS and stained with 0.4% AO/PI to check the viability of Countstar Rigel S2 (Countstar, Shanghai).

For SnRNA‐seq, frozen tissue samples were cut into pieces <0.5 cm and homogenized using a glass Dounce tissue grinder (Sigma, #D8938). Tissues were homogenized 25 times with pestle A and 25 times with pestle B in ice‐cold nuclei EZ lysis buffer. The nucleus pellets were collected by centrifugation, washed, and resuspended with nuclei suspension buffer (NSB; consisting of 0.01% BSA and 0.1% RNase inhibitor (Clontech, #2313A)). Nuclei suspensions were then filtered through a 35 µm cell strainer (Corning‐Falcon, #352 235) and counted. A final concentration of 1000 nuclei µL^−1^ was used for loading on a 10x channel.

### 10x Library Preparation and Sequencing

Prepared single‐cell or nucleus suspensions were then used for 10x library preparation and sequencing. Beads with unique molecular identifier (UMI) and cell barcodes were loaded to near saturation, ensuring that each cell was paired with a bead in a Gel Beads‐in emulsion (GEM). Following exposure to cell lysis buffer, polyadenylated RNA molecules hybridized to the beads. The beads were retrieved into a single tube for reverse transcription. During cDNA synthesis, each cDNA molecule was tagged at the 3′end (corresponding to the 5′end of a messenger RNA transcript) with UMI and cell label indicating its cell of origin. Subsequently, the 10x beads underwent second‐strand cDNA synthesis, adaptor ligation, and universal amplification. Sequencing libraries were prepared using randomly interrupted whole‐transcriptome amplification products to enrich the 3′ end of the transcripts linked with the cell barcode and UMI. All remaining procedures including the library construction, were performed according to the standard manufacturer's protocol (Chromium Single Cell 3ʹ v3.1). The sequencing libraries were quantified using a High Sensitivity DNA Chip (Agilent) on a Bioanalyzer 2100 and assessed with the Qubit High Sensitivity DNA Assay (Thermo Fisher Scientific). The E14.5 libraries were sequenced on Nova 6000(Illumina) on platformed of Suzhou Dynamic Biosystems Co., Ltd (Suzhou, China), as well as P3 and P22 libraries were sequenced on Nova Xplus (Illumina) on platform of Majorbio Co., Ltd (Shanghai, China) using 2 × 150 chemistry. ScRNA‐seq and snRNA‐seq data files have been deposited in the NCBI Sequence Read Archive (SRA) database under BioProject accession number PRJNA1171138.

### Bioinformatic Analysis

For bioinformatics, reads were processed using the Cell Ranger V7.1.0 pipeline with default and recommended parameters. FASTQ files generated from Illumina sequencing output were aligned to the mouse GRCm39 genome, using the STAR algorithm. Gene‐Barcode matrices were created for each individual sample by counting UMIs and filtering out non‐cell associated barcodes. Finally, a gene‐barcode matrix containing the barcoded cells and gene expression counts was generated. This output was then imported into the Seurat (v4.1.1) R toolkit for quality control and downstream analysis of scRNA‐seq or snRNA‐seq data. All functions were executed with default parameters unless specified otherwise. Matrices were curated to filter out low‐quality cells based on a standard set of three quality control criteria: 1) the number of detected transcripts, quantified by unique molecular identifiers; 2) the number of detected genes; and 3) the percentage of reads mapping to mitochondrial genes, which were identified as being outside the mean value plus or minus twofold the standard deviation. The calculation of mitochondrial gene expression was performed using the PercentageFeatureSet function from the Seurat package.

The normalized data (NormalizeData function in Seuratpackage) was subjected to the extraction of a subset of variable genes. These variable genes were identified while controlling for the strong relationship between their variability and average expression levels. Subsequently, data were integrated from different samples by identifying “anchors” between datasets using the Find Integration Anchors and Integrate Data functions in the Seurat package. Principal component analysis (PCA) was then conducted. The resulting clusters were visualized on a 2D map generated with UMAP (Uniform Manifold Approximation and Projection).

Cells were clustered using graph‐based clustering of the PCA‐reduced data with the Louvain Method, following the computation of a shared nearest neighbor graph. For sub‐clustering, the same procedure of scaling, dimensionality reduction, and clustering to the specific set of data, typically focusing on one type of cell was applied. For each cluster, the Wilcoxon Rank‐Sum Test was utilized to identify significantly differentially expressed genes when comparing the remaining clusters. Both SCINA and known marker genes were employed to ascertain cell types.

### Differential Expression Genes Analysis

To identify DEGs between two different samples, the FindMarkers function in Seurat, utilizing a likelihood ratio test was used. DEGs with log2FC ≥ 0.25 and *p*‐value <0.05 were considered to be significantly different expressed genes. In addition, functional‐enrichment analysis of GO and KEGG were carried out by Goatools (https://github.com/tanghaibao/Goatools) and Python scipy software, respectively.

### Cell–Cell Communication Networks

The cell–cell communication networks between astrocytes and other nervous cell clusters were performed using CellphoneDB statistical analysis, a computational approach predicting CCIs by ligand‐receptor interactions analysis.

### Primary Astrocytes Culture

Mouse primary astrocytes were prepared as previously described with minor modifications.^[^ ^54^ ^]^ Briefly, brain tissues from mice at P1, P6, P8, and P20 were dissected and carefully stripped of their meninges. Subsequently, the tissues were rinsed with a PBS solution containing 2% glucose and digested with ACCUTASE (STEMCELL Technologies) for 10 min at 37 °C to achieve a single‐cell suspension. The cell suspension was then cultured in primary astrocyte medium (iCell Bioscience Inc) on a plate coated with Matrigel (Corning, #356 230) at 37 °C for ≈10 days. Medium was replaced every 3 days during this period. Microglia and oligodendrocytes were separated by vigorously shaking the flasks at 200 r.p.m. for a continuous 24 h at 37 °C. The remaining cells were pure astrocytes. They were cultured for an additional 5–7 days until confluent.

### Neural Stem and Progenitor Cells Culture

Mouse neural stem and progenitor cells were prepared using NeuroCult Proliferation Kit (STEMCELL Technologies, 05702) according to the manufacturer's protocol. Briefly, brain tissues were digested with ACCUTASE (STEMCELL Technologies) and dissociated into a single‐cells. The cell suspension was then cultured in the NeuroCult Proliferation Kit containing 20 ng mL^−1^ Human Recombinant EGF (STEMCELL Technologies, 78006.1) on plate coated with Matrigel (Corning, #356 230) at 37 °C.

### Antibodies

The primary antibodies used were listed as below: Anti‐PRKCBP1/RACK7(A302‐089A, Bethyl), Anti‐histone H3K27ac (ab4729, Abcam), Anti‐histone H3K4me1 (39297, Active motif), Anti‐histone H3K4me3 (#9727, Cell signaling technology), Anti‐histone H3K36me3 (#4909, Cell signaling technology), Anti‐histone H3K27me3 (#9733, Cell signaling technology), Anti‐Histone H3 (ab18521, Abcam), Anti‐Histone H4 (#13919, Cell signaling technology), Anti‐Ezh2(#5246, Cell signaling technology), Anti‐EED (#85322, Cell signaling technology), Anti‐SUZ12 (#3737, Cell signaling technology), Anti‐RBBP4 (20364‐1‐AP, Proteintech), Anti‐Lamin B1 (66095‐1‐Ig, Proteintech), Anti‐GFAP (16825‐1‐AP, Proteintech), Anti‐GFAP (#80788, Cell signaling technology), Anti‐NeuN (#24307, Cell signaling technology), Anti‐Nestin (ab6142, Abcam), Anti‐OLIG2 (#65915, Cell signaling technology), Anti‐S100 beta (ab52642, Abcam).

### Tissue Section

Postnatal mice were anesthetized and perfused with 20 mL of PBS. The brain tissues were excised and fixed in a 4% Paraformaldehyde fix solution (G1101, Servicebio) at 4 °C for 20 h. Subsequently, the tissue was rinsed 3 times with PBS, embedded in paraffin, and sectioned to a thickness of 4 µm. The tissue sections were then subjected to Nissl staining, immunofluorescence, and multiplex immunofluorescence imaging. Tissue sections and Nissl staining were performed by Servicebio.

### Immunofluorescence

Tissue sections were deparaffinized with xylene and rehydrated through a series of decreasing concentrations of ethanol. Antigen retrieval was performed using the Improved Citrate Antigen Retrieval Solution (P0083, Beyotime Biotechnology). To block endogenous peroxidase activity, the sections were incubated with H_2_O_2_ in darkness for 15 min. Following three washes with PBST buffer, the sections were blocked with 10% serum in PBST buffer for 1 h at room temperature and stained with primary antibodies overnight at 4 °C. After three additional washes with PBST buffer, the slides were incubated with Goat anti‐Rabbit IgG (H+L) Cross‐Adsorbed Secondary Antibody conjugated with Alexa Fluor 555 (A‐21428, Invitrogen) and DAPI solution(1 mg mL^−1^,1:1000) (C0060, Solarbio) for 1 hour at room temperature in dark. All antibodies were diluted in 5% serum in PBST buffer. Stained slides were washed again in PBST and mounted with Fluoromount Aqueous Mounting Medium (F4680, Sigma). Images were captured using a research slide scanner (VS200, Olympus).

Immunofluorescence and imaging of P1, P7, and P22 brain slides with anti‐RACK7 antibodies were performed by Zhiwei Biotechnology (Zhengzhou) Co., Ltd. Images were acquired using a research slide scanner (Fusion, Akoya).

Quantitative analysis of tissue section image was performed by TissueGnostics Aisa Pacific limited (Beijing, China). Briefly, the cell density of nucleus area per cell, area per cell, and expression per cell were quantified using StrataQuest software (version 7.1.129, TissueGnostics GmbH, Vienna, Austria).

For primary astrocytes, the immunofluorescence procedure was performed as previously described.^[^ ^8^ ^]^

### Multiplex Immunofluorescence (mIF) Imaging

mIF and imaging were performed by Zhiwei Biotechnology (Zhengzhou) Co., Ltd. The mIF was performed using the Opal 6‐plex manual detection kit (Akoya, NEL861001KT) according to the manufacturer's protocol. Primary antibodies and their corresponding fluorescent dyes are depicted in Figure S2d (Supporting Information). Stained slides were washed with TBST buffer and mounted with Anti‐Fade Fluorescence Mounting Medium (ab104135, Abcam). Images were acquired using a research slide scanner (Fusion, Akoya).

### Immunoblotting

Mice tissues or primary cells were collected, washed with cold PBS solution, and lysed using RIPA buffer (P0013B, Beyotime Biotechnology). Protein lysates were denatured, loaded onto an SDS‐PAGE gel, and transferred to an NC membrane (Millipore). The membranes were blocked with 5% no‐fat milk in TBST buffer at room temperature for 1 hour and then immunoblotted with primary antibodies diluted in 5% BSA in TBST buffer overnight at 4 °C. The membranes were then incubated with either Goat anti‐Rabbit IgG Secondary Antibody HRP conjugated (L3012, Signalway antibody) or Goat anti‐Mouse IgG Secondary Antibody HRP conjugated (L3032, Signalway antibody) for 1 hour at room temperature. Visualization was performed using the Smart‐ECL Super kit (S32500, Smart‐life sciences), and images were captured with the Bio‐Rad ChemiDoc Touch Imaging System.

### Behavioral Tests

All mouse behavioral tests were performed in the Department of Laboratory Animal Science, Fudan University. Mice aged 3 to 4 weeks, of both sexes, were used for all behavioral studies. The experiments were conducted during the light phase of the light/dark cycle, between 9:00 a.m. and 5:00 p.m. Prior to testing, mice were habituated to the testing room for at least 30 min. In between tests with different animals, all equipment was cleaned with ethanol. Statistical analyses were performed using two‐way ANOVA tests. The detailed procedures are as follows:

### Open Field

Spontaneous motor activity was measured using an automated open‐field system (SuperFlex, Omnitech). A mouse was placed in the center of a plexiglass enclosure (42 cm × 42 cm square base with 31 cm‐high walls) and its activity was recorded using infrared light beams over a 30‐min period. In the data analysis, the floor was virtually divided into 16 identical squares, with the central 4 squares designated as a distinct zone to assess anxiety‐like behavior.

### Rotarod

Motor coordination was assessed on an accelerating rotarod. Mice were placed on the rotarod (KY‐ROTD, Kuoyunbio) for three consecutive days, with three trials per day spaced 30 min apart. For each trial, the rotarod accelerated from 5 to 40 rpm over a period of 300s, and the latency to fall was recorded.

### EEG (Electroencephalogram) Recordings

EEG recording and data analysis were conducted by Bio‐signal technologies as follows. Mice were anesthetized and mounted in a stereotaxic apparatus. Screw electrodes were inserted into the skulls of the mice to measure cortical EEG using the following coordinates: +2 mm Bregma, +1 mm midline for first recording electrode; +2 mm Bregma, −1 mm midline for second recording electrode; −2 mm Bregma, −1 mm midline for a reference electrode; and −2 mm Bregma, +1 mm midline for a ground electrode. After recovery for 6 days, the mice (maintained under a 12 h light/dark cycle) were subjected to EEG recordings for 47 hours in the Homecage of Medusa acquisition system (Bio‐signal Technologies, China). Data were acquired using a tethered data acquisition system with a sampling rate of 1000 Hz. The EEG data were visually screened to identify potential convulsive seizure events using EDFbrowser.

### Chromatin IP and Sequencing (ChIP‐seq)

ChIP‐seq assays were performed as previously described.^[^ ^8^ ^]^ Briefly, primary astrocytes were cross‐linked with 1% formaldehyde (Sigma) for 10 min and stopped with 125 mm glycine for 5 min. Cells were then lysed with ChIP lysis buffer (50 mm Hepes pH 7.5, 500 mm NaCl, 1 mm EDTA, 1% Triton, 0.1% Na‐deoxycholate, 0.05% SDS,1 mm PMSF, 1 mm DTT and protease inhibitors) and sonicated using a Qsonica sonicator. Chromatin samples were incubated with 5 µg of primary antibodies as listed and immobilized onto Dynabeads Protein A and G (1002D and 1004D, Invitrogen). Notably, the H3K27me3 antibody was co‐incubated with spike‐in chromatin (53 083, Active motif) and spike‐in antibody (61686, Active motif) to normalize according to the manufacturer's protocol. The bound fractions were washed three times with the lysis buffer, three times with radioimmunoprecipitation assay buffer (50 mm Hepes, 300 mm LiCl, 1 mm EDTA, 0.5% NP‐40, 0.7% Na‐deoxycholate), and once with 50 mm NaCl in Tris‐EDTA. The DNA fractions were eluted and reverse cross‐linked in the elution buffer (50 mm tris‐HCl pH 8.0, 10 mm EDTA, and 1% SDS) at 65 °C for 6 h. Following treatment with RNase A and proteinase K, DNA was purified using the PCR Extraction Kit (28104, Qiagen) and quantified using the Qubit dsDNA HS Assay kit (Q32851, Thermofisher). ChIP‐seq sequencing libraries were prepared using the VAHTS Universal DNA Library Prep Kit for Illumina V3 (ND607‐02, Vazyme) according to the manufacturer's instructions. The completed ChIP‐seq libraries were qualified by Bioanalyzer 2200(Agilent) and sequenced at the Azenta life science and MINGMA TECHNOLOGIES using Illumina NovaSeq 6000 instrument. The raw and analyzed ChIP‐seq data files have been deposited in the NCBI Gene Expression Omnibus (GEO) database under ID code: GSE270581.

For bioinformatics analysis, ChIP‐seq reads were aligned to a concatenated genome sequence of mouse (mm10) using Bowtie2.3.4.3. Specifically, H3K27me3 spike‐in ChIP‐seq reads were aligned to both the mouse (mm10) and Drosophila (dm 6) concatenated genome sequences using Bowtie2.^[^ ^55^ ^]^ Peaks were called using MACS2 (2.1.1).^[^ ^56^ ^]^ Heatmaps were generated with plotProfile/plotHeatmap functions from deep‐Tools (2.5.7).^[^ ^57^ ^]^

### Co‐Immunoprecipitation (Co‐IP)

Forebrain tissues from eight WT neonatal mice were utilized for the Co‐IP assay. The tissues were washed with cold PBS, digested with ACCUTASE (STEMCELL Technologies) for 10 min at 37 °C, and dispersed into a single‐cell suspension. The cell pellet was treated with hypotonic buffer (10 mm Tris‐HCl pH 7.3, 10 mm KCl, 1.5 mm MgCl_2_) and homogenized by a Douncing pestle (loose type) for 25 to 30 strokes. The resulting cell suspension was centrifugation at 3500 rpm for 15 min at 4 °C to remove the cytoplasm. The pellets were resuspended in high salt buffer (20 mm Tris‐HCl pH 7.3, 400 mm KCl, 1.5 mm MgCl_2_, 0.2 mm EDTA, 25% glycerol, 1 mm β‐ME, and protease inhibitors) and stirred for one hour at 4 °C. The mixture was centrifuged at 13 500 rpm for 30 min at 4 °C to obtain the nuclear extract. The nuclear extract was then dialyzed against a dialysis buffer (20 mm Tris‐HCl pH 7.3, 100 mm KCl, 0.2 mm EDTA, 20% glycerol, 10 mm β‐ME, and 1 mm PMSF) overnight at 4 °C. Following dialysis, the extract was centrifuged at 12000 rpm for 30 min at 4 °C. The supernatant was incubated with 2 µg anti‐RACK7 or control IgG (Cell signal technology, #2729), and subsequently mixed with rProtein A/G Magarose Beads (Smart Lifesciences, SM005002) for 8 h. The bound proteins were washed 5 times with wash buffer (50 mm Tris‐HCl pH 7.9, 100 mm KCl, 5 mm MgCl_2_, 0.2 mm EDTA, 10% glycerol, 0.1% NP‐40, 3 mm β‐ME) and then subjected to immunoblots.

### In Vitro RACK7 Pulldown

Exogenous human HA‐tagged RACK7 full‐length were expressed and purified from *Sf9* insect cells as previously described.^[^ ^8^ ^]^ The dialyzed nuclear extract prepared from the forebrain of wild‐type mice or 2 µg recombinant PRC2 complex protein (31 387, Active motif) was incubated with exogenous RACK7 protein in binding buffer (20 mm Tris‐HCl pH 7.3, 150 mm KCl, 0.2 mm EDTA, 20% glycerol, 0.1% NP‐40, and 1 mm PMSF) for 6 hours at 4 °C, followed by incubation with anti‐HA infinity beads (Smart Lifesciences, SA068005) for an additional 2 h. The bound proteins were washed 5 times with wash buffer (50 mm Tris‐HCl pH 7.9, 150 mm KCl, 5 mm MgCl_2_, 0.2 mm EDTA, 10% glycerol, 0.1% NP‐40) and then subjected to immunoblots.

### RNA‐Seq and Data Analysis

mRNA capture and sequencing were performed by Azenta life science. For bioinformatics analysis, the mRNA sequencing reads were mapped to mouse reference genome (GRCm38) using the software Hisat2. Differentially expressed genes were calculated by the ballgown program (version 2.12.0) and DESeq2 program with cutoffs as follows: *p*‐value < 0.05, and foldchange ≥1.5. Two biological repeats were used for each cell line. The raw and analyzed RNA‐seq data files have been deposited in the NCBI GEO database under ID code: GSE270582.

### Rack7 Transgene Rescue

Lentiviruses carrying the *Rack7* transgene (Gene ID 228 880) and negative control were purchased from Genechem (Shanghai, China) and used to infect both the WT and *Rack7*‐cKO primary astrocytes according to the manufacturer's instructions. One‐week post‐infection, cells were harvested, and total RNA was extracted using TRIzol Reagent (Invitrogen, #15596018CN). Complementary DNAs were synthesized with the PrimeScript II Reverse Transcriptase Kit with gDNA Eraser (TaKaRa, #RR047A). The synthesized cDNA, gene‐specific primers, and ChamQ Universal SYBR qPCR Master Mix (Vazyme, #Q711) were combined and subjected to the LightCycler 480 II Real‐Time PCR System (Roche). The gene‐specific primers used in this study were synthesized by Biosune, and the sequences are as below (5′ to 3′; F, forward; R, Reverse): *Wnt2*: F‐ CTCTCGGTGGAATCTGGCTC, R‐ CCTGTAGCTCTCATGTACCACC; *Wnt2b*: F‐ AAGAGGCTTAAGGATGCCCG, R‐ GGAATCTCCGAACAGCCGTG; *Pax6*: F‐ CACCAGACTCACCTGACACC, R‐ TCACTCCGCTGTGACTGTTC; *Wnt9a*: F‐ GCCTACTTCGGGCTGACG, R‐ GGTCGCAGGCCTTGTAGTG; *Rpl19*: F‐GGAAGGGTACTGCCAATGCT, R‐TCCATGAGGATGCGCTTGTT. The significant of all the primers was validated by melting curve analyzation.

### ATAC‐Seq

WT and *Rack7*‐cKO primary astrocytes were collected and lysed with lysis buffer (10 mm Tris‐HCl pH 7.4, 10 mm NaCl, 3 mm MgCl_2,_ and 0.5% NP‐40). ATAC‐seq was performed using TruePrep DNA Library Prep Kit V2 for Illumina (Vazyme, TD501) according to the manufacturer's instructions. The completed ATAC‐seq libraries were qualified by Bioanalyzer 2200 (Agilent) and sequenced at the MINGMA TECHNOLOGIES using Illumina NovaSeq 6000 instrument. For bioinformatics analysis, ATAC‐seq reads were aligned to a concatenated genome sequence of mouse (mm10) using bowtie2, and peaks were called using MACS2(2.1.1).

### Statistical Analysis

GraphPad Prism (9.0.0) software was used for data analysis. For graphs, data are shown as mean ± SD, the statistical significance of the dataset in Figure 5h was determined using one‐way ANOVA, and other datasets were determined by two‐way ANOVA; ∗*p* ≤ 0.05, ∗∗*p* ≤ 0.01, ∗∗∗*p* ≤ 0.001, ∗∗∗∗*p* ≤ 0.0001, n.s, = not significant.

## Conflict of Interest

The authors declare no conflict of interest.

## Author Contributions

R.G. conceived and supervised the project; F.J. carried out all experiments and bioinformatics analysis with assistance from B.W., S.H., T.T., Y.Z., B.X., and R.G.; B.W., L.D., and Y.Z. helped with mouse genotyping and behavior tests; R.G. provided assistance with bioinformatics analysis and contributed additional experimental data during the revision of the manuscript, S.H. helped with molecular biology experiments; T.X advised on IHC, mIF, scRNA‐seq, and behavior experimental designs; Y.L. provided suggestions to the mouse model establishment and primary cell culture; P.Z. provided discussions and offered funding support; F.J. and R.G wrote the manuscript with input from all authors.

## Supporting information



Supporting Information

## Data Availability

The data that support the findings of this study are available from the corresponding author upon reasonable request.
