# Supplementary material for: RACK7 Interacts with PRC2 Complex to Regulate Astrocyte Development
Source: Adv Sci (Weinh). 2025 Mar 24;12(19):2416350. doi: 10.1002/advs.202416350 (PMC12097129; doi:10.1002/advs.202416350)
Supplement: Supplementary file 1 — Supporting Information [file ADVS-12-2416350-s001.docx]

Supporting Information

RACK7 interacts with PRC2 complex to regulate astrocyte development

*Fangfang Jiao, Tianxiang Tang, Bowen Wang, Shengfei He, Yue Zhang, Li Dong, Bo Xu, Ying Liu, Ping Zhu, and Rui Guo**


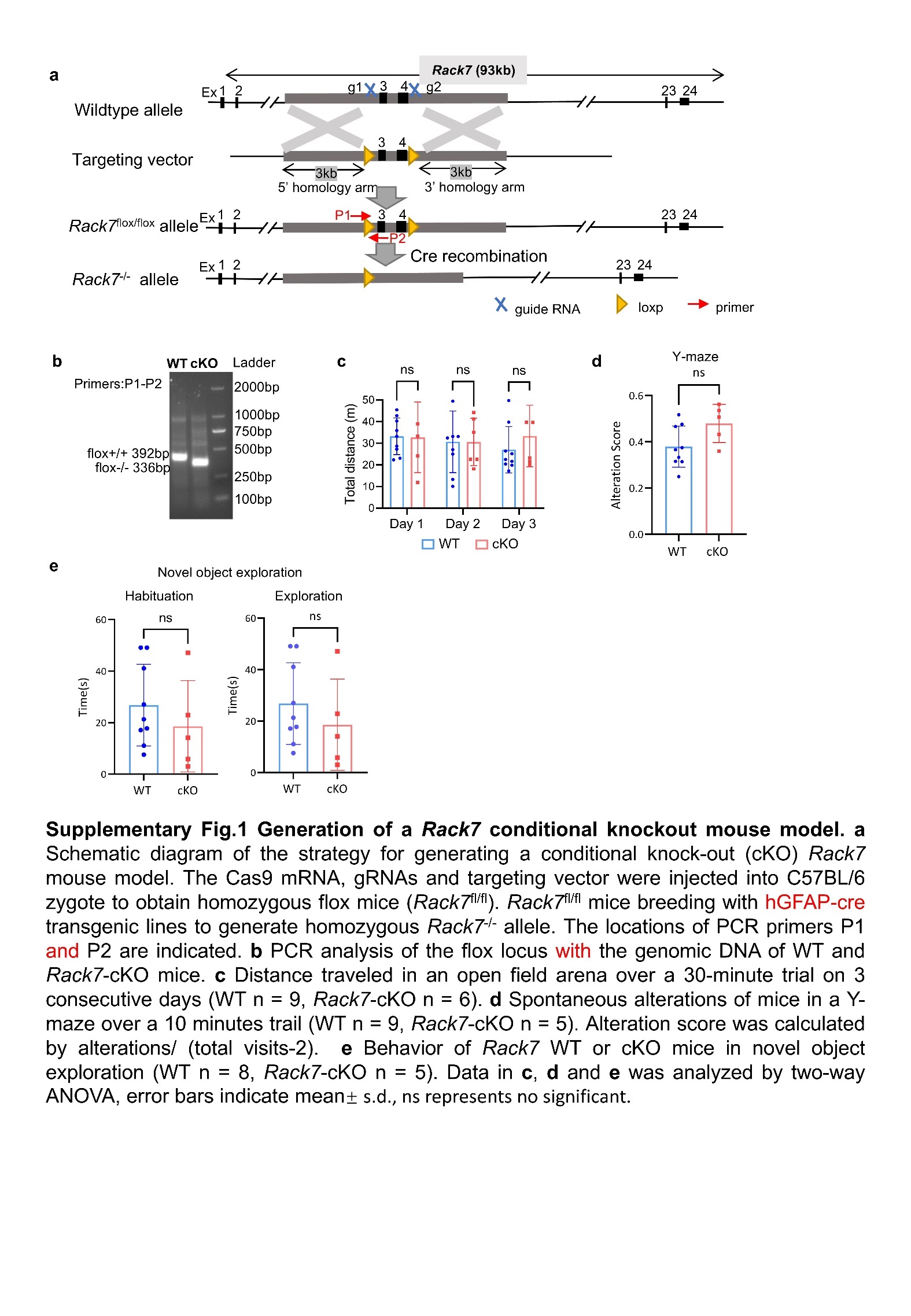


**Figure S1 Generation of a *Rack7* conditional knockout mouse model.** **a**) Schematic diagram of the strategy for generating a *Rack7-* cKO mouse model. The Cas9 mRNA, gRNAs and targeting vector were injected into C57BL/6 zygote to obtain homozygous flox mice (*Rack7* ^flox/flox^). *Rack7* ^flox/flox^ mice breeding with hGFAP-cre transgenic lines to generate homozygous *Rack7* ^-/-^ allele. The locations of PCR primers P1 and P2 are indicated. **b**) PCR analysis of the flox locus with the genomic DNA of WT and *Rack7*-cKO mice. **c**) Distance traveled in an open field arena over a 30-minute trial on 3 consecutive days (WT, *n* = 9; *Rack7*-cKO, *n* = 6). **d**) Spontaneous alterations of mice in a Y-maze over a 10 minutes trail (WT, *n* = 9; *Rack7*-cKO, *n* = 5). Alteration score was calculated by alterations/ (total visits-2). **e**) Behavior of *Rack7*-WT or cKO mice in novel object exploration (WT, *n* = 8; *Rack7*-cKO, *n* = 5). Data in **c**, **d** and **e** was analyzed by two-way ANOVA, error bars indicate mean ±SD, ns represents no significant.


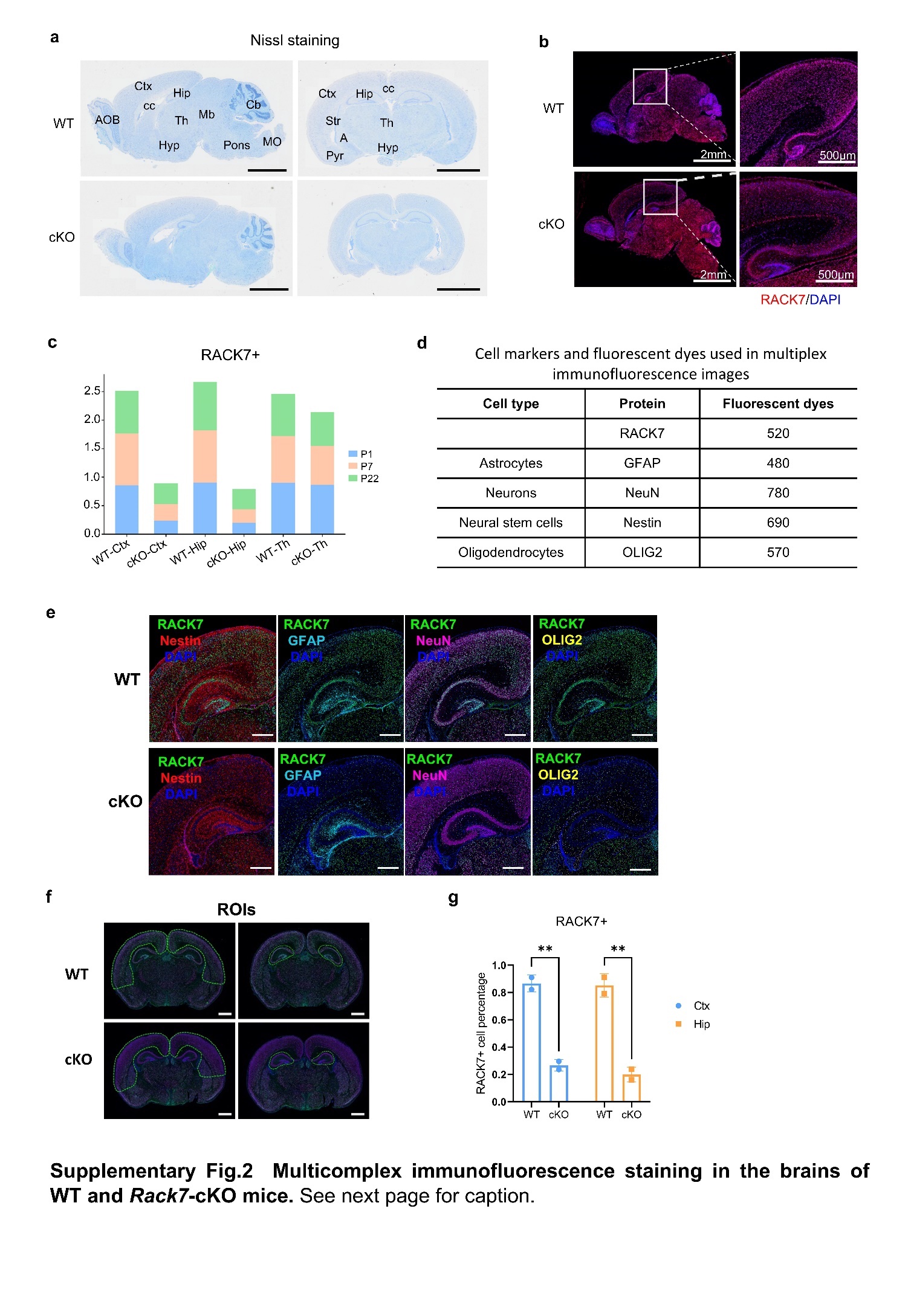


**Figure S2 Multicomplex immunofluorescence staining in the brains of WT and *Rack7*-cKO mice. a)** Nissl staining of WT and *Rack7*-cKO coronal brain sections at P30. Regions are labeled as follows: A, amygdala; AOB, accessory olfactory bulb; Cb,cerebellum; cc, corpus callosum; Ctx, cerebral cortex; Hip, hippocampus; Hyp, hypothalamus; Mb, midbrain; MO, medulla oblongata; Pyr, pyriform cortex; Str, striatum; Th, thalamus. Scale bars= 2 mm. **b)** IF staining of RACK7 in WT and *Rack7*-cKO brain slides at P7. **c)** Quantitative analysis of RACK7-positive (+) cells in Ctx, Hip and Th of WT and *Rack7*-cKO brain slides at P1, P7 and P22. **d)** Cell markers and fluorescent dyes used in mIF images. **e)** Determination of ROIs in WT and *Rack7*-cKO brain slides. **f)** Quantitative analysis of RACK7-positive (+) cells in Ctx and Hip of WT and *Rack7*-cKO brain slides. Data are presented as the mean ±SD from two biological repeats. Statistical significance was determined using two-way ANOVA, ***p* represents *p* ≤ 0.01.


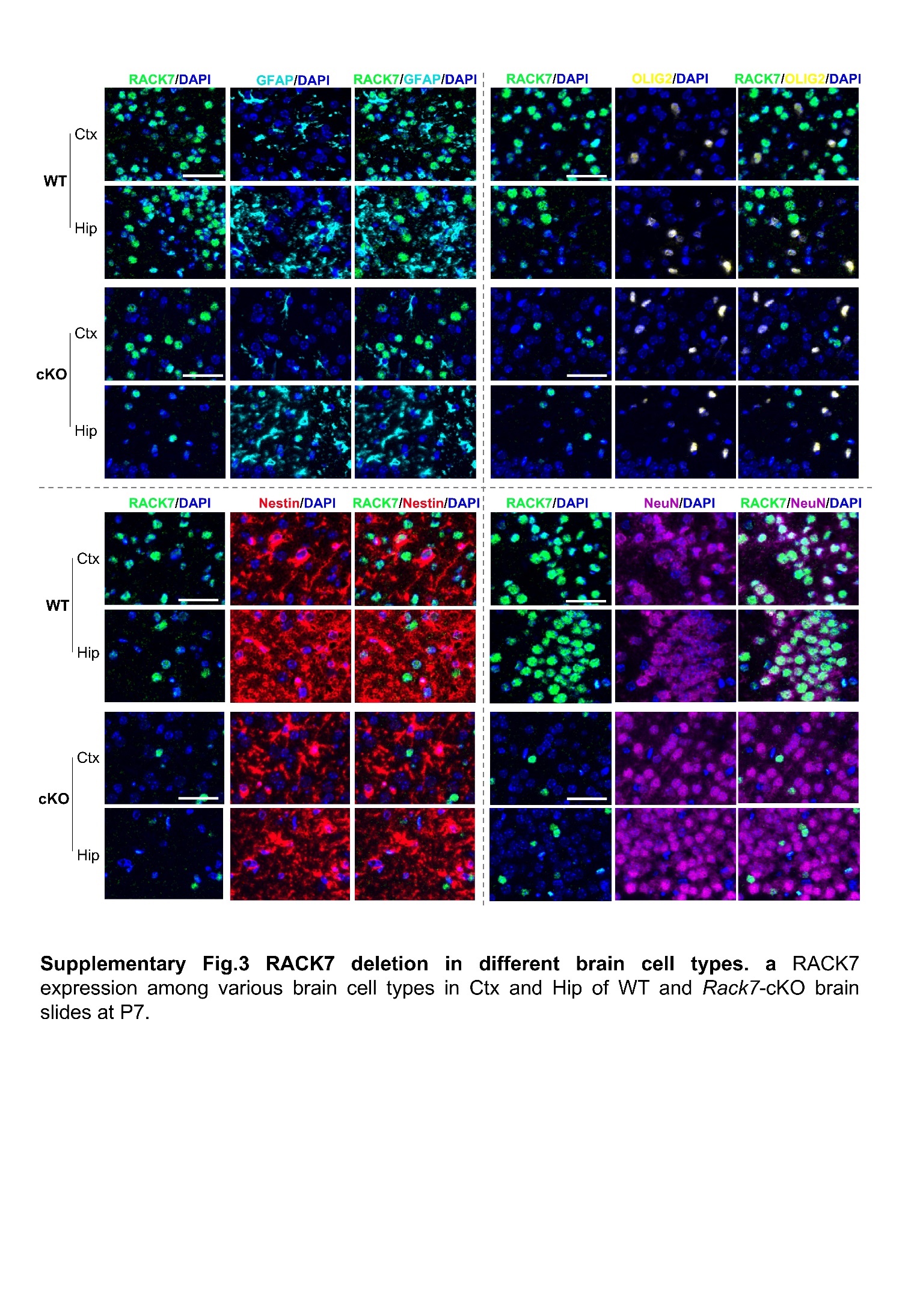


**Figure S3 RACK7 deletion in different brain cell types.** RACK7 expression among various brain cell types in Ctx and Hip of WT and *Rack7*-cKO brain slides at P7.


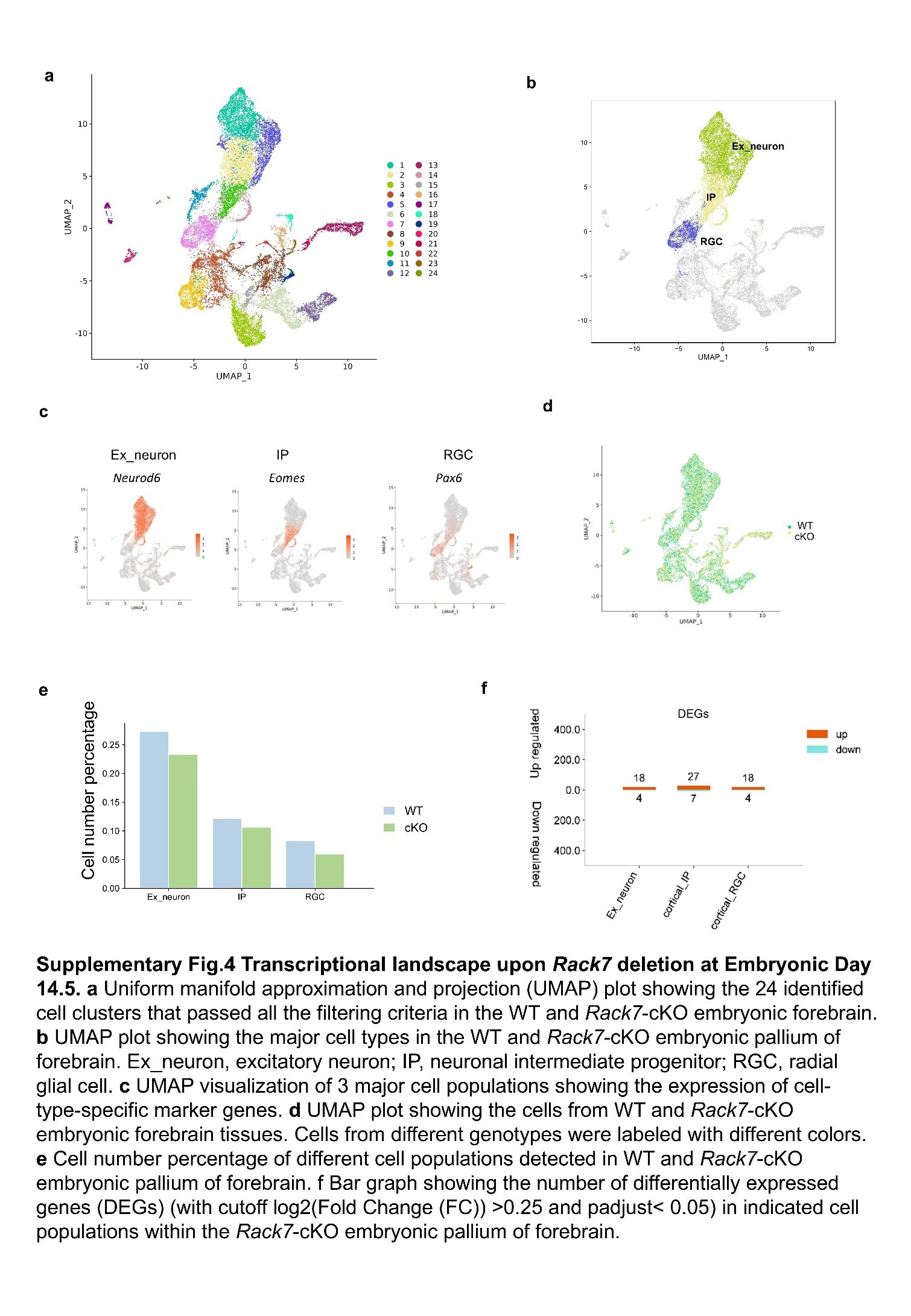


**Figure S4 Transcriptional landscape upon *Rack7* deletion at Embryonic Day 14.5. a)** Uniform manifold approximation and projection (UMAP) plot showing the 24 identified cell clusters that passed all the filtering criteria in the WT and *Rack7*-cKO embryonic forebrain. **b)** UMAP plot showing the major cell types in the WT and *Rack7*-cKO embryonic pallium of forebrain. Ex_neuron, excitatory neuron; IP, neuronal intermediate progenitor; RGC, radial glial cell. **c)** UMAP visualization of 3 major cell populations showing the expression of cell-type-specific marker genes. **d)** UMAP plot showing the cells from WT and *Rack7*-cKO embryonic forebrain tissues. Cells from different genotypes were labeled with different colors. **e)** Cell number percentage of different cell populations detected in WT and *Rack7*-cKO embryonic pallium of forebrain. **f)** Bar graph showing the number of DEGs (with cutoff log2(FC) >0.25 and *p-*adjust< 0.05) in indicated cell populations within the *Rack7*-cKO embryonic pallium of forebrain.


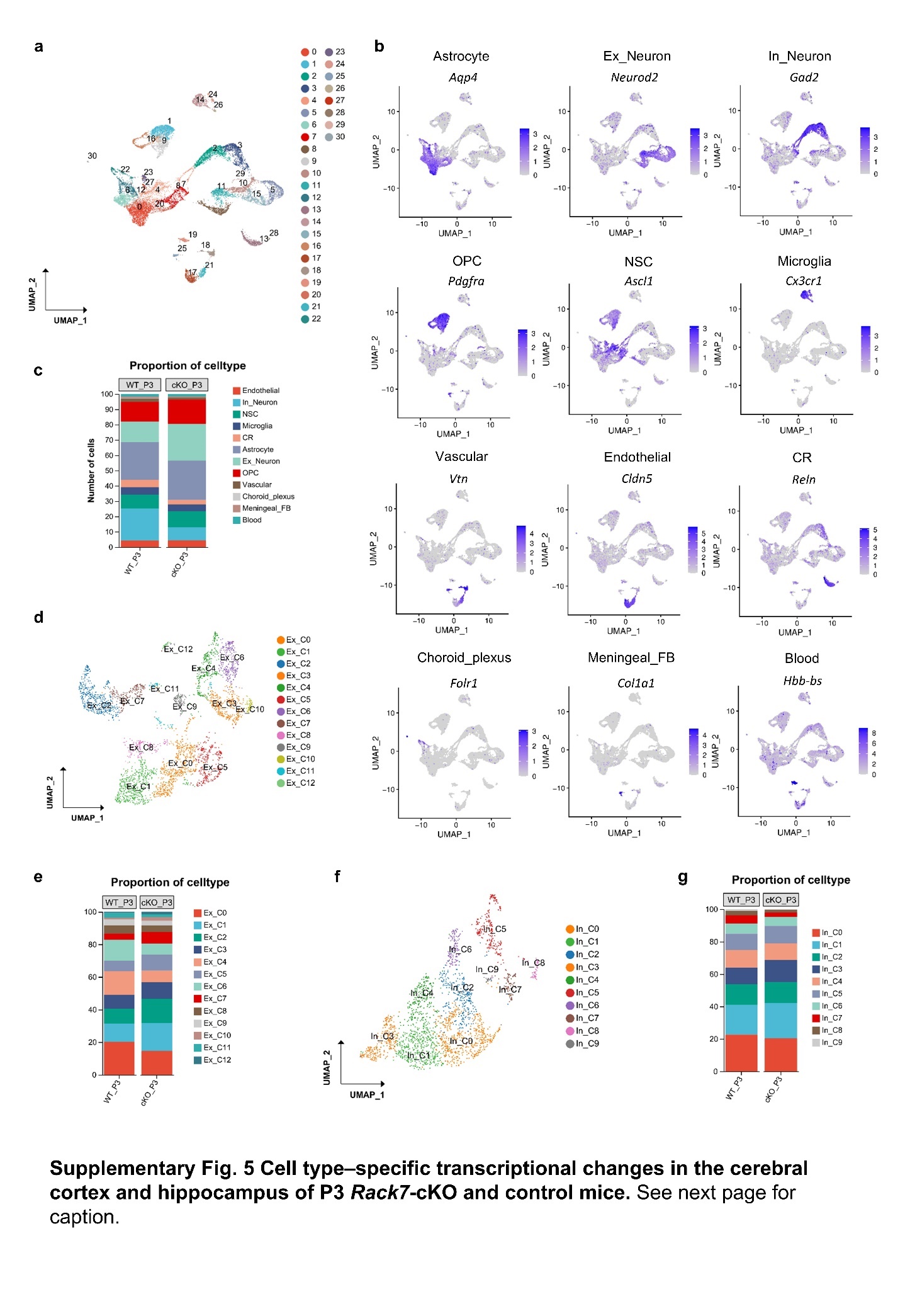


**Figure S5 Cell type–specific transcriptional changes in the cerebral cortex and hippocampus of P3 *Rack7*-cKO and control mice.** **a)** UMAP plot showing the 30 identified cell clusters that passed all the filtering criteria. **b)** UMAP visualization of 12 major cell populations showing the expression of cell-type-specific marker genes. **c)** Percentage of cell types presented in Figure 3a. **d)** UMAP plot showing the sub-clusters within the excitatory neuron population. **e)** Percentage of each sub-cluster within the excitatory neuron population. **f)** UMAP plot showing the sub-clusters from inhibitory neuron population. **g)** Percentage of each sub-cluster within the inhibitory neuron population.


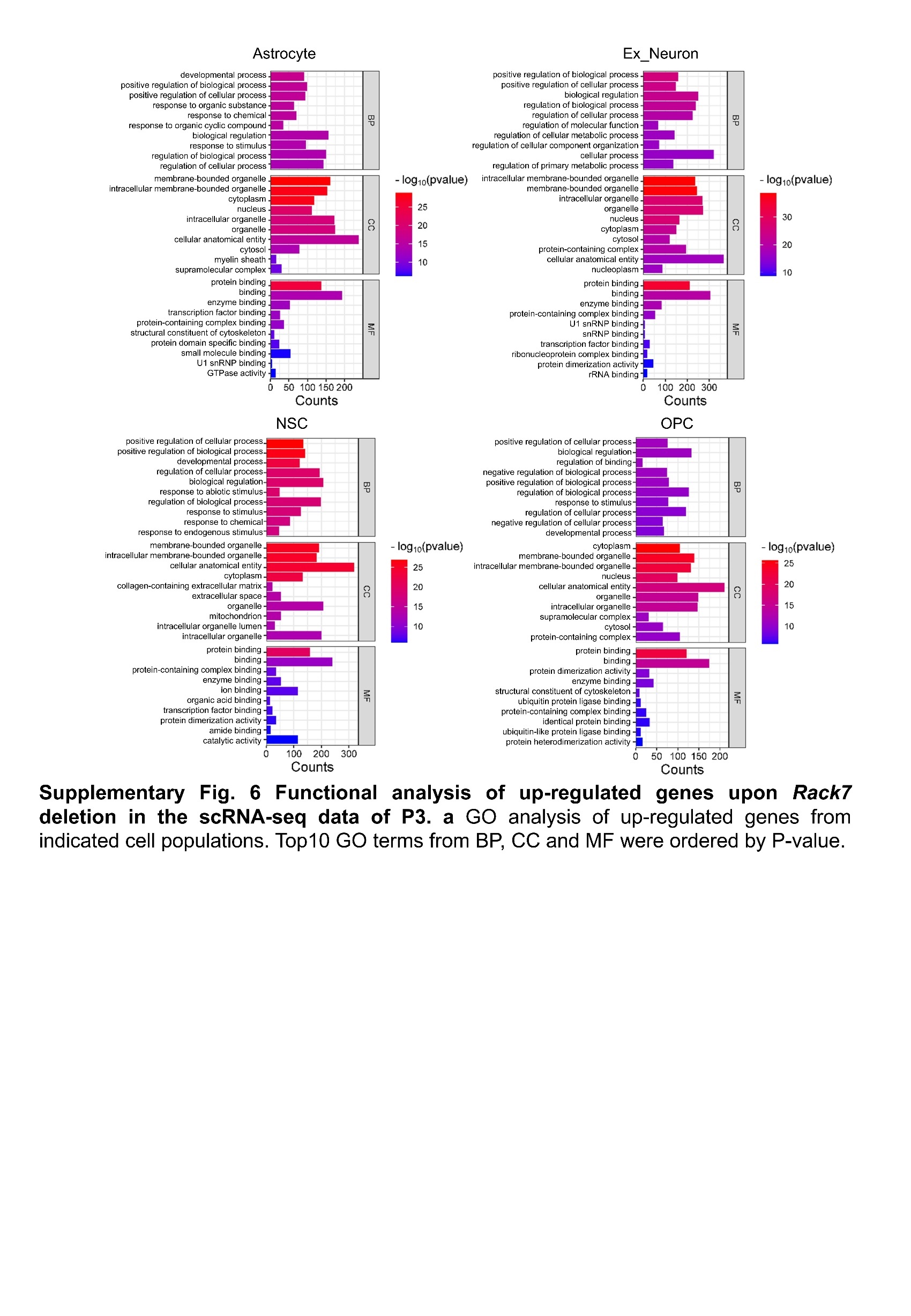


**Figure S6 Functional analysis of up-regulated genes upon *Rack7* deletion in the scRNA-seq data of P3.** GO analysis of up-regulated genes from indicated cell populations. Top10 GO terms from BP, CC and MF were ordered by *p*-value.


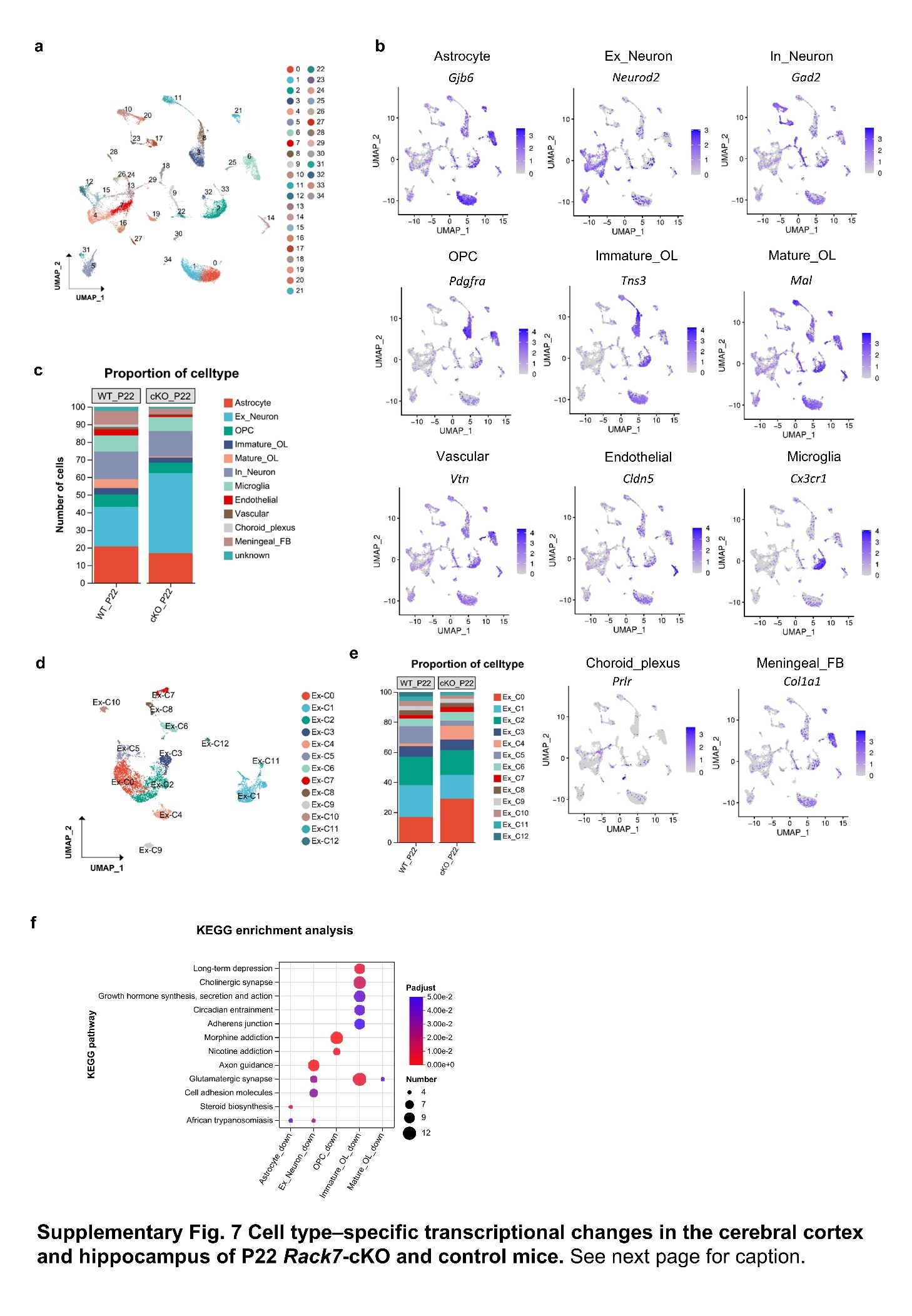


**Figure S7 Cell type–specific transcriptional changes in the cerebral cortex and hippocampus of P22 *Rack7*-cKO and control mice.** **a)** UMAP plot illustrating the 34 identified cell clusters that passed all filtering criteria. **b)** UMAP visualization of 11 major cell populations showing the expression of cell-type-specific marker genes. **c)** Percentage of each cell type in Figure 4a. **d)** UMAP plot showing the sub-clusters of the excitatory neuron population. **e)** Percentage of each sub-clusters among the excitatory neuron population. **f**) KEGG pathway analysis of the down-regulated genes from indicated cell populations. Top10 KEGG terms were ordered by *p*-adjust. The KEGG terms that *p*-adjust ≥ 0.05 are not shown.


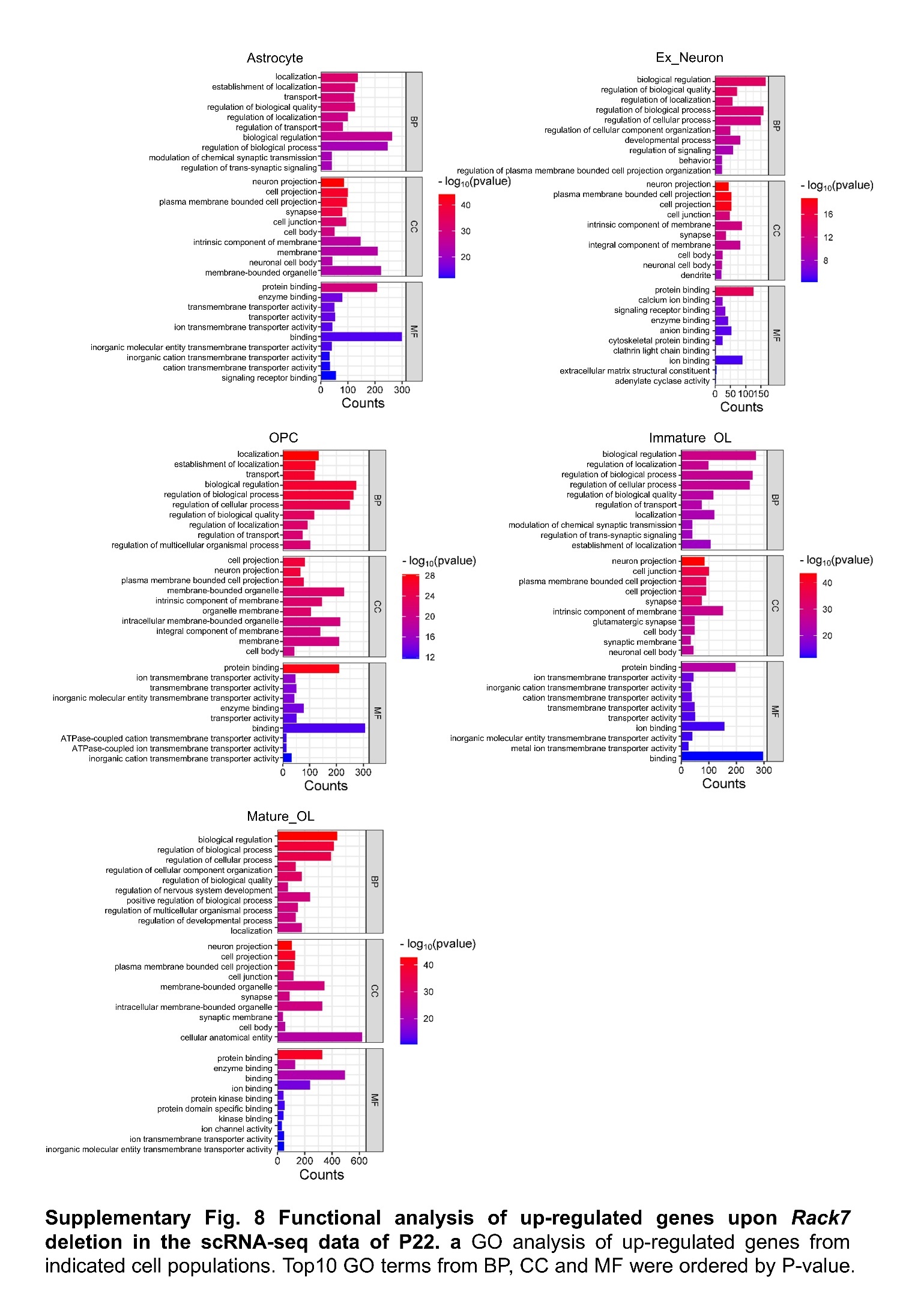


**Figure S8 Functional analysis of up-regulated genes upon *Rack7* deletion in the scRNA-seq data of P22.** GO analysis of up-regulated genes from indicated cell populations. Top10 GO terms from BP, CC and MF were ordered by *p*-value.


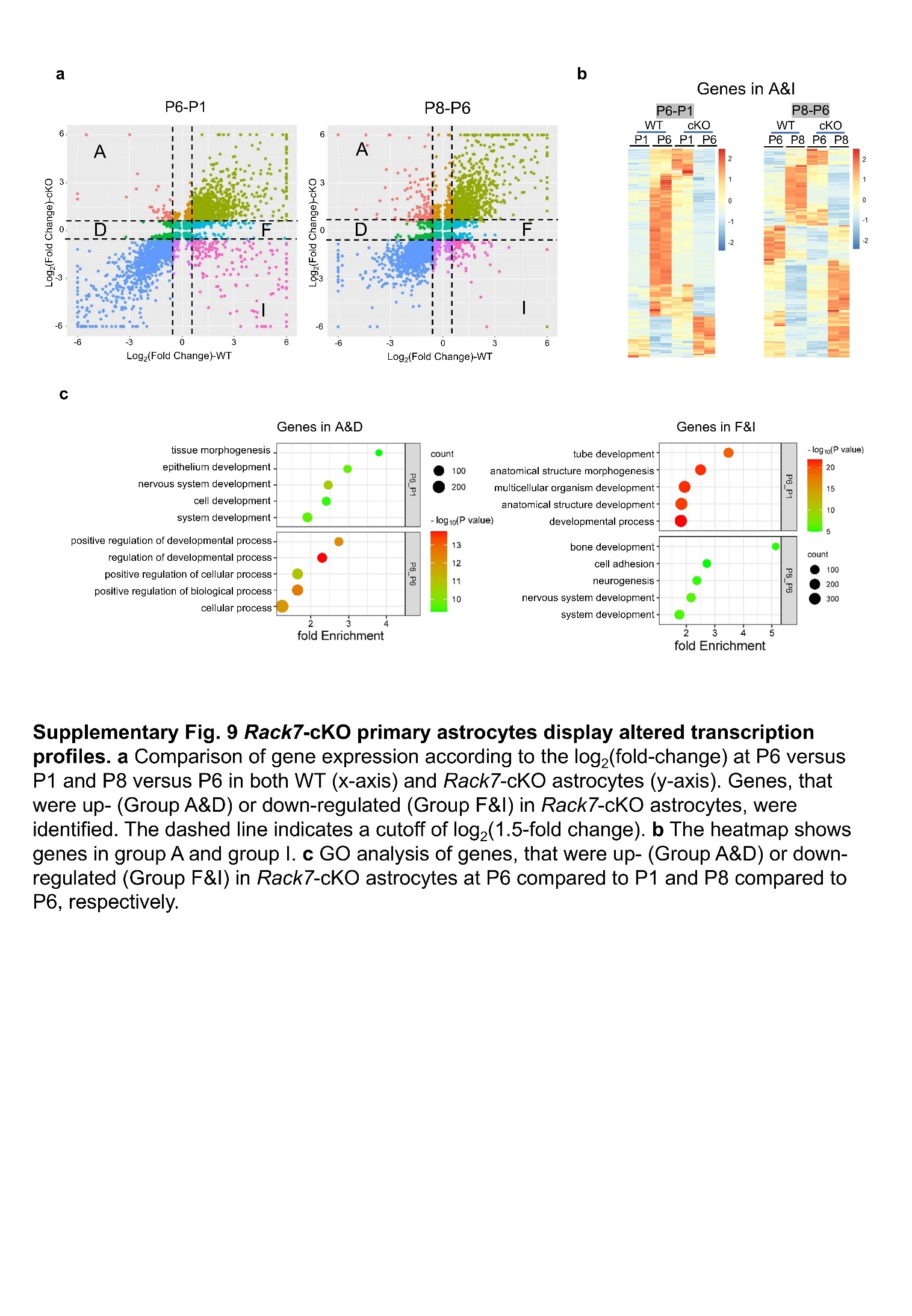


**Figure S9** ***Rack7*-cKO primary astrocytes display altered transcription profiles. a)** Comparison of gene expression according to the log_2_(FC) at P6 versus P1 and P8 versus P6 in both WT (x-axis) and *Rack7*-cKO astrocytes (y-axis). Genes, that were up- (Group A&D) or down-regulated (Group F&I) in *Rack7*-cKO astrocytes, were identified. The dashed line indicates a cutoff of log_2_(1.5-FC). **b)** The heatmap shows genes in group A and group I. **c** GO analysis of genes, that were up- (Group A&D) or down-regulated (Group F&I) in *Rack7*-cKO astrocytes at P6 compared to P1 and P8 compared to P6, respectively.


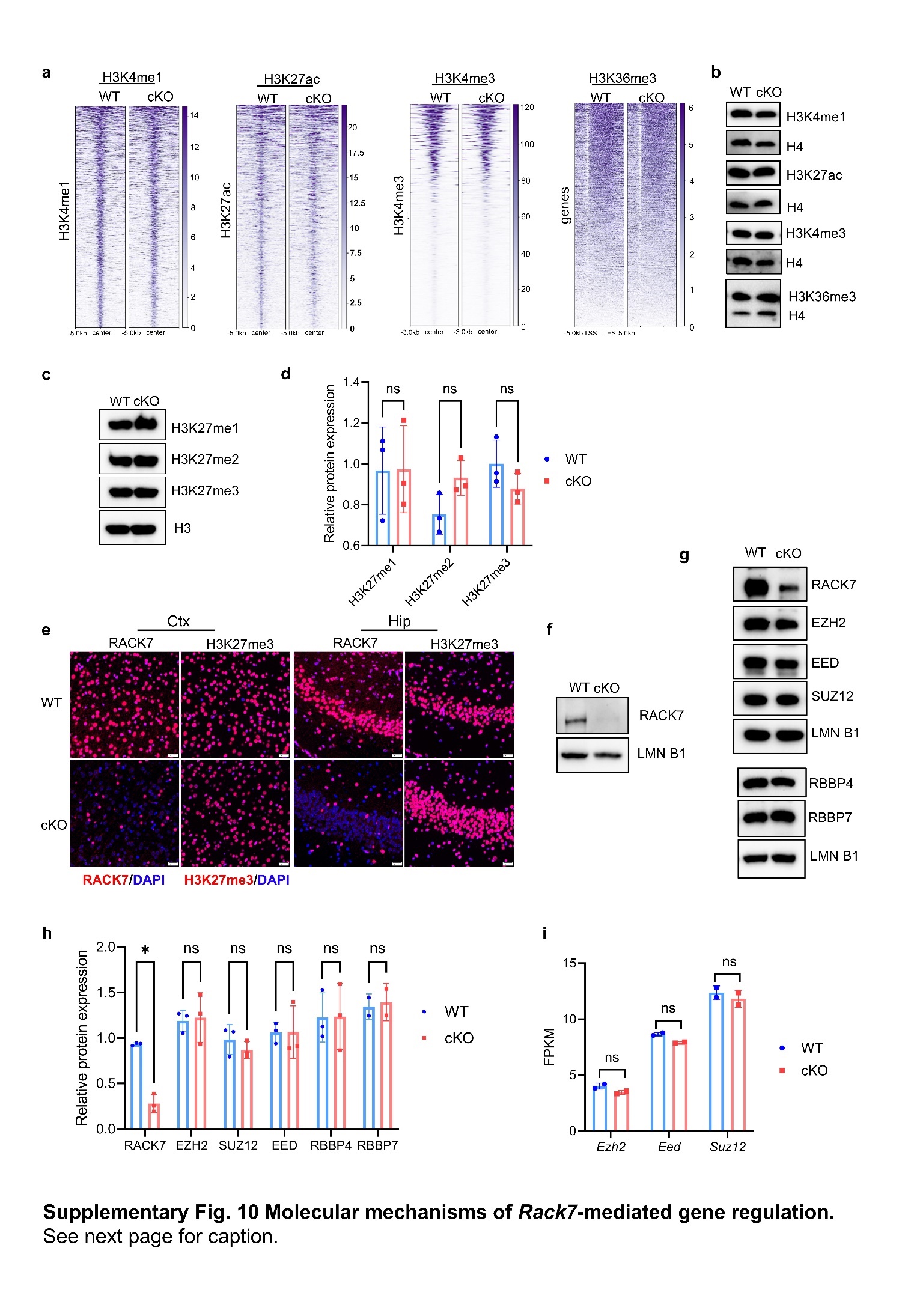


**Figure S10** **Molecular mechanisms of *Rack7*-mediated gene regulation.** **a)** Heatmap analysis of H3K4me1, H3K27ac, H3K4me3 and H3K36me3 ChIP-seq signals in WT and *Rack7*-cKO primary astrocytes. **b)** Immunoblots of histone H3K4me1, H3K27ac, H3K4me3 and H3K36me3 in WT and *Rack7*-cKO primary astrocytes. **c)** Immunoblots of histone H3K27me1, H3K27me2 and H3K27me3 in WT and *Rack7*-cKO primary astrocytes. **d)** Densitometric quantification of immunoblot results of H3K27me1, H3K27me2 and H3K27me3 from three biological repeats. **e)** IF staining of RACK7 and H3K27me3 in Ctx and Hip of WT and *Rack7*-cKO brain slides. Scale bars = 20µm. **f)** Immunoblots of RACK7 in WT and *Rack7*-cKO primary NSCs and NPCs. **g)** Immunoblots of protein level of RACK7 and PRC2 components. **h)** Densitometric quantification of immunoblot results of RACK7 and PRC2 components from three biological repeats. **i)** FPKM of PRC2 core components in RNA-seq data of P6 astrocytes. Data in **d**, **h**, **i** are presented as the mean ± SD, statistical significance was determined using two-way ANOVA, **p* represents *p*≤0.05, ns represents no significant.


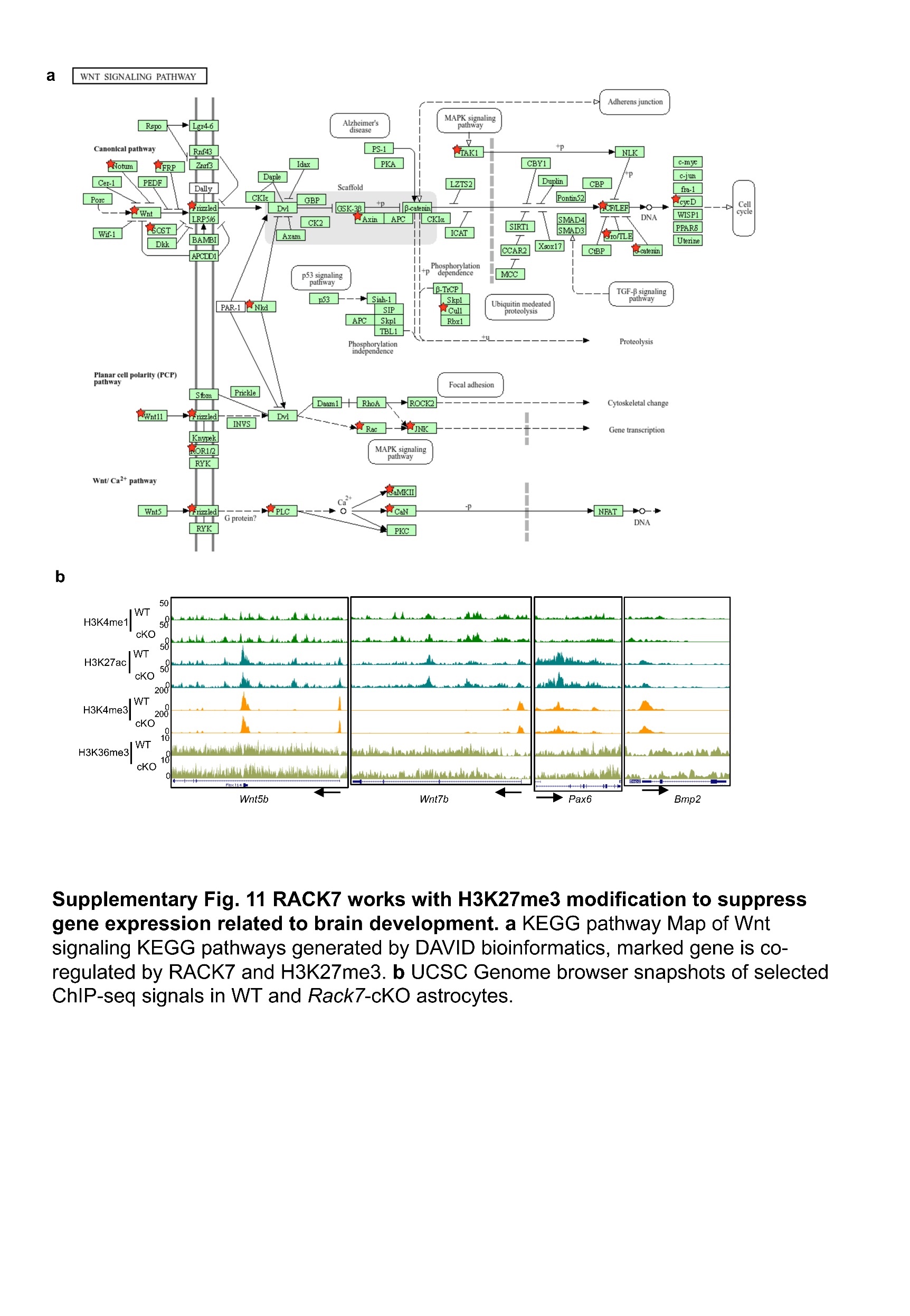


**Figure S11** **RACK7 works with H3K27me3 modification to suppress gene expression related to brain development.** **a)** KEGG pathway Map of Wnt signaling KEGG pathways generated by DAVID bioinformatics, marked gene is co-regulated by RACK7 and H3K27me3. **b)** UCSC Genome browser snapshots of selected ChIP-seq signals in WT and *Rack7*-cKO astrocytes.


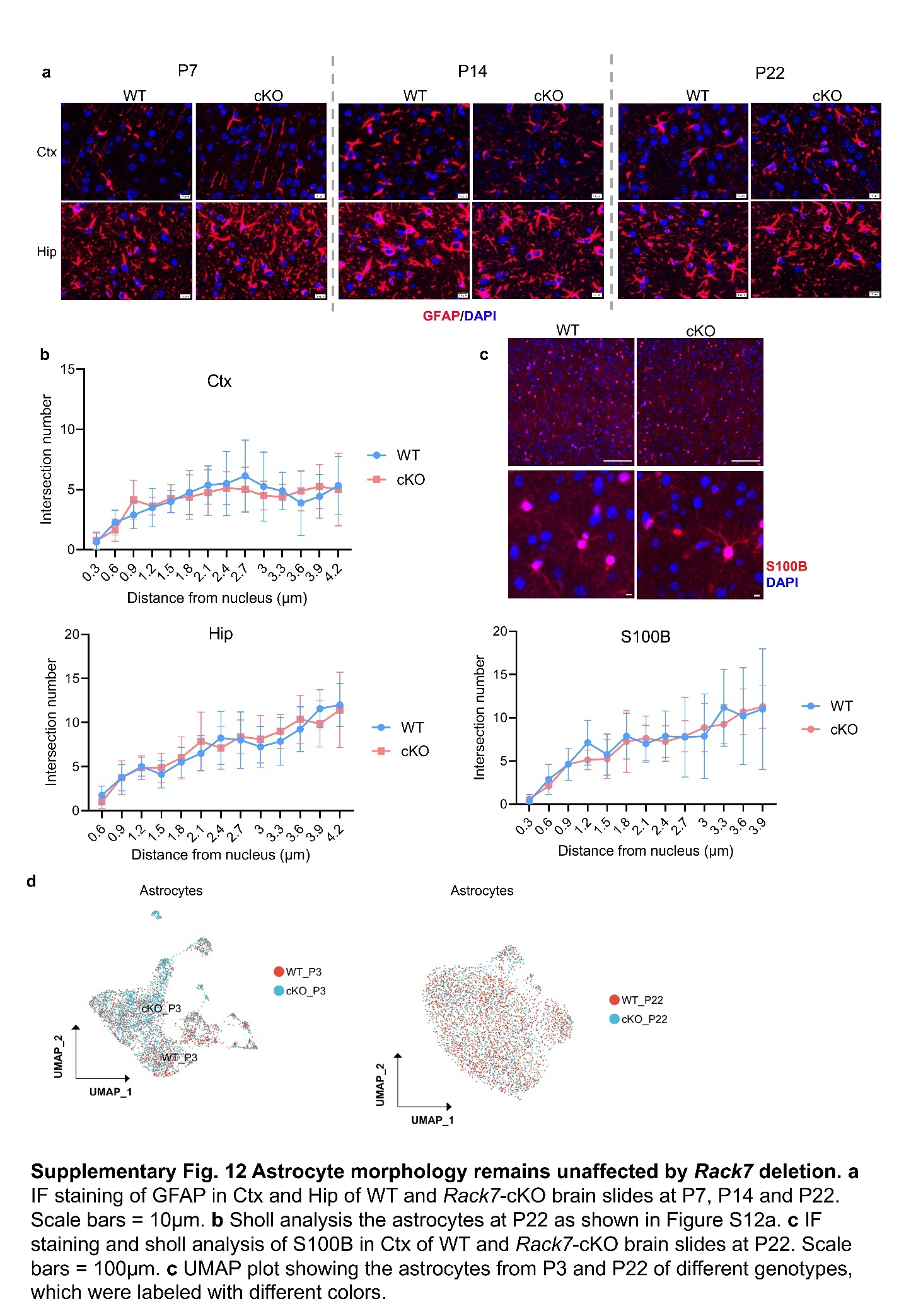


**Figure S12 Astrocyte morphology remains unaffected by *Rack7* deletion. a)** IF staining of GFAP in Ctx and Hip of WT and *Rack7*-cKO brain slides at P7, P14 and P22. Scale bars = 10µm. **b)** Sholl analysis of the IF staining images of GFAP at P22. Cell numbers: *n*=8. **c**) IF staining and sholl analysis of S100B in Ctx of WT and *Rack7*-cKO brain slides at P22. Scale bars = 100µm. Cell numbers: *n*=8. **d)** UMAP plot showing the astrocytes from P3 and P22 of different genotypes, which were labeled with different colors.
